# Engineering asymmetric solvation structures for synergistically boosted quasi-solid thermocells

**DOI:** 10.1039/d6sc03942a

**Published:** 2026-06-22

**Authors:** Wentao Lin, Shuo Niu, Shukai Wu, Chao Fang

**Affiliations:** a Sustainable Energy and Environment Thrust, The Hong Kong University of Science and Technology (Guangzhou) Guangzhou Guangdong 511400 China chaofang@hkust-gz.edu.cn

## Abstract

Quasi solid-state thermocells (QTECs) based on the thermogalvanic effect offer a promising route for directly converting abundant low-grade heat into electricity. Introducing a single solvent into hydrogel electrolytes, a common strategy to enhance thermopower, often yields a marginal solvation entropy difference between redox ions and provides limited gains in ion transport. To break this longstanding trade-off, we present a simple yet highly effective co-solvent strategy that employs trimethyl phosphate and ethylene glycol to construct hybrid hydrogel electrolytes. This approach synergistically enlarges solvation entropy differences of redox ions, amplifies the concentration gradient across the thermocell, and enhances redox ion transport through the hydrogel network. The resulting hydrogel electrolyte achieves superior thermoelectrochemical performance and demonstrates efficient harvesting of low-grade heat even at sub-zero temperatures. Advanced characterization techniques, integrated with molecular simulations, elucidate that the enhanced thermoelectrochemical performance originates from co-solvent engineered asymmetric solvation structures. This work demonstrates targeted, additive-free modulation of the solvation environment in thermogalvanic hydrogels as a practical strategy to significantly enhance thermoelectrochemical performance.

## Abstract 2

A co-solvent strategy employing trimethyl phosphate and ethylene glycol in hydrogel thermocells overcomes the trade-off between thermopower and ion transport through asymmetric solvation structures for efficient low-grade heat harvesting.

## Graphical abstract

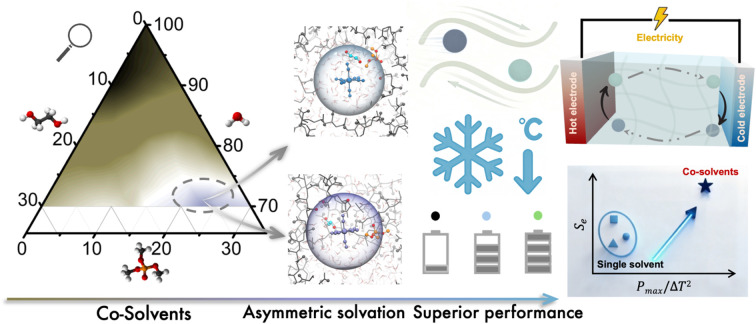

## 1 Introduction

The pursuit of effective and sustainable waste heat utilization techniques is crucial for global energy conservation and achieving carbon neutrality goals.^1–5^ At present, approximately 60% of the world's energy is ultimately wasted as heat, most of which is low-grade heat (<100 °C).^6,7^ Thermocells (TECs), which operate based on the thermogalvanic effect, have attracted growing attention due to their promising capacity for harvesting waste low-grade heat.^8–12^ A typical TEC consists of two electrodes separated by an electrolyte containing redox couple ions. When a temperature gradient (Δ*T*) is applied between the electrodes, an electric potential difference develops through temperature-driven redox reactions, thus allowing nonintermittent heat-to-electricity conversion. Recently, quasi solid-state TECs (QTECs) based on hydrogel electrolytes (thermogalvanic hydrogels, THs) have attracted considerable interest as a practical and scalable platform to harvesting low-grade thermal energy, particularly for self-powered wearable electronics.^13,14^ These systems are recognized for their considerable thermopower (known as the Seebeck coefficient, *S*_e_), which often exceeds 1 mV K^−1^, alongside advantages such as low costs.^15–19^

The solvation structure of redox ions is a critical factor governing the thermoelectrochemical performance of THs, as it directly determines the entropy change (Δ*S*_rc_) of the redox reaction.^12,17,20–22^ In the case of a widely used redox couple, Fe(CN)_6_^4−/3−^,^23–27^ the high-valence ion experiences stronger charge–dipole interactions with the surrounding solvent molecules, which leads to a more ordered solvation shell than that of Fe(CN)_6_^3−^. Such a difference in the local solvation structure generates disparity in the temperature-dependent response of the solvation free energy between two redox ions, essentially solvation entropy. This term contributes to the overall entropy change of a redox reaction, as captured in the expansion of Seebeck coefficient:^28^1
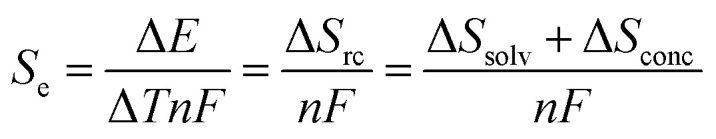
where Δ*E* is the potential difference developed across Δ*T*, *n* is the number of electrons transferred, and *F* is Faraday's constant. This expression decouples Δ*S*_rc_ into contributions from ion solvation (Δ*S*_solv_) and concentration (Δ*S*_conc_). In particular, Δ*S*_solv_ arises from the difference in the ratio of the redox ions' activity coefficients between the two electrodes (equivalently, the solvation entropy difference). On the other hand, Δ*S*_conc_ scales with the difference in the ratio of the redox ions' concentrations between the electrodes. Importantly, the Δ*S*_conc_ term itself is affected by ion solvation, as solvation dictates the solubility of redox ions.^18,29–31^ Therefore, even minor changes in the ordering and dynamics of the local ion solvation environment can significantly influence the thermoelectrochemical performance.^17,32–34^

A high *S*_e_ is desirable for practical applications, particularly for powering IoT sensors or wearable devices, where efficient utilization of low-grade heat under small Δ*T* is indispensable for sustainable operation. Recent research has demonstrated that *S*_e_ can be effectively enhanced by introducing organic solvents.^29,31,35^ For example, a study on an ethylene glycol (EG)/polyacrylamide (PAAm)-based TH demonstrated through spectral characterization that EG modifies the solvation structures and solubility of Fe(CN)_6_^4−/3−^ ions.^31^ Consequently, *S*_e_ was boosted from 1.30 to 2.04 mV K^−1^ with this alteration. In another study with a bacterial cellulose-based TH, the introduction of propylene glycol induced selective reconstruction of the Fe(CN)_6_^4−^ solvation shell, which contributed to a high *S*_e_ of 2.30 mV K^−1^.^29^ Despite these improvements, a fundamental challenge persists for practical deployment of single-solvent THs. The inverse relationship between key electrolyte properties, such as *S*_e_ and ionic conductivity *σ*, severely compromises the output power density, thereby limiting the energy-harvesting capability of QTECs.

Herein, we develop a co-solvent engineering strategy employing trimethyl phosphate (TMP) and EG within a polyacrylamide (PAAm) hydrogel to construct asymmetric solvation structures and enable efficient ion transport. Although EG is known to modulate solvation structures, its capacity to compete with water molecules in the primary solvation shell of Fe(CN)_6_^4−/3−^ is mitigated by the resemblance of hydroxyl groups to water. To address this constraint, we introduced TMP, an unexplored solvent molecule in thermocells that features a highly polar phosphate ester group, to more effectively reconstruct the solvation structures of Fe(CN)_6_^4−/3−^. The combination of TMP and EG was found to synergistically enlarge the solvation entropy difference and amplify concentration gradients of the redox ions as captured by eqn (1). This effect, together with enhanced ion–polymer interaction and salt dissociation, delivers exceptional thermoelectrochemical performance in the co-solvent hydrogel electrolytes that persists even at sub-zero temperatures. Our work provides a practical strategy and new insights for the design of high-performance QTECs *via* the rational, additive-free manipulation of the solvation environment.

## 2 Results

### 2.1 Characteristics of co-solvent hydrogel electrolyte

**Fig. 1:**
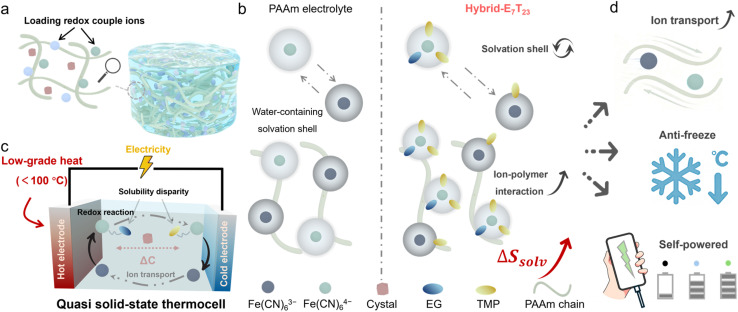
Design strategy of co-solvent hydrogel electrolyte. (a) Schematic of the Hybrid-E_*x*_T_*y*_ assembly. (b) Comparison of the ion solvation structure and ion–polymer interactions between the PAAm and Hybrid-E_7_T_23_ electrolytes. (c) Co-solvent induced crystallization of Fe(CN)_6_^4−^ and its role in enhancing the thermogalvanic effect. (d) Illustration of enhanced ion transport and anti-freeze properties of Hybrid-E_7_T_23_, along with its potential applications.

The design strategy for enhancing the thermoelectrochemical performance of hydrogel electrolyte is illustrated in Fig. 1. A co-solvent (EG/TMP) hydrogel electrolyte (Fig. 1a) was fabricated *via* a standard two-step procedure as shown in Fig. S1. Initially, acrylamide, initiators, and crosslinkers were dissolved in ultrapure water and stirred to form a homogeneous solution. The solution was subsequently injected into a glass plat mold and heated to form a pure PAAm hydrogel. Next, EG solvent, TMP solvent, and K_3_Fe(CN)_6_–K_4_Fe(CN)_6_ salt were incorporated into the PAAm hydrogel *via* solution exchange. This yields a hydrogel electrolyte containing both aqueous and organic solvents. To investigate the relationship between intermolecular interactions and thermoelectrochemical performance, a series of parallel electrolytes were prepared with varying EG-to-TMP ratios. For clarity, the hydrogel electrolyte systems were denoted as “Hybrid-E_*x*_T_*y*_” (short for “Hybrid-(EG)_*x*_(TMP)_*y*_”), where *x* and *y* denote the volume percentages of EG and TMP in the solution phase, respectively.

The continuous operation of the Hybrid-E_*x*_T_*y*_-based QTEC is sustained by the thermal voltage generated between the cold and hot sides under Δ*T*. The magnitude of this voltage, characterized by *S*_e_, is determined by the solvation entropy difference between two redox ions and their concentration gradients between the hot and cold electrodes, as illustrated by eqn (1). The Hybrid-E_*x*_T_*y*_ system demonstrates excellent thermoelectrochemical performance as compared to both pure PAAm electrolyte and single-solvent hydrogel electrolytes. The enhancement in the solvation entropy difference mainly originates from co-solvent (EG and TMP) induced restructuring of the solvation environment of redox ions. Due to the preferential interaction between Fe(CN)_6_^4−^ and co-solvents, a greater number of solvent molecules are incorporated into the solvation shell as compared to Fe(CN)_6_^3−^. This results in more asymmetric solvation structures to effectively enlarge their solvation entropy difference (Fig. 1b). Meanwhile, the accessibility of coordination sites along the PAAm backbone to Fe(CN)_6_^4−/3−^ is modulated by the co-solvents. The induced differential polymer–ion interactions further amplify the solvation entropy difference between redox ions.

The restructuring of the solvation environment also leads to a marked alteration in the thermodynamic solubility of redox ions, as depicted in Fig. 1c. Fe(CN)_6_^4−^ exhibits significantly lower solubility relative to Fe(CN)_6_^3−^, primarily due to the different ion–solvent interactions that tend to stabilize one oxidation state rather than the other.^31^ Upon application of Δ*T*, an uneven concentration distribution of redox ions across the thermogalvanic cells will be developed. The resulting concentration gradient contributes directly to the *S*_e_, a contribution that is further amplified by the reversible dissolution/crystallization of Fe(CN)_6_^4−^ ions between the cold and hot electrodes. Apart from the substantial enhancement in *S*_e_, the Hybrid-E_7_T_23_ system demonstrates improved ion transport, superior anti-freeze properties, and the capability to form a self-powered QTEC, as shown in Fig. 1d. Therefore, the synergistic interplay between the co-solvents enables Hybrid-E_7_T_23_ to achieve exceptional thermoelectrochemical performance.

### 2.2 Thermoelectrochemical performance

**Fig. 2:**
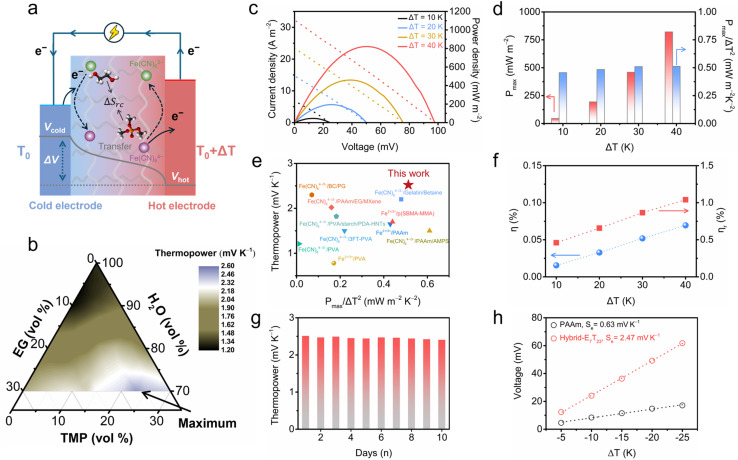
Thermoelectrochemical performance of hybrid electrolytes. (a) Schematic representation of the heat-to-electricity conversion mechanism of QTECs. (b) Ternary phase diagram of *S*_e_ in Hybrid-E_*x*_T_*y*_ with varying volume ratios of EG, TMP, and water. (c) Output current–voltage–power curves of Hybrid-E_7_T_23_ at various Δ*T*, and (d) corresponding *P*_max_ and *P*_max_/Δ*T*^2^ values. (e) Comparison of *S*_e_*versus P*_max_/Δ*T*^2^ for various QTECs. (f) Thermal energy conversion efficiency of Hybrid-E_7_T_23_. (g) Temporal variation of *S*_e_ of Hybrid-E_7_T_23_ under continuous operation for 10 days. (h) The *S*_e_ of the PAAm electrolyte and Hybrid-E_7_T_23_ at sub-zero temperatures.

The Hybrid-E_*x*_T_*y*_-based QTEC operates on the thermogalvanic effect, wherein a temperature difference across the electrolyte drives reversible redox reactions that establish a potential difference between the electrodes. As illustrated in Fig. 2a, at the hot electrode, Fe(CN)_6_^4−^ undergoes oxidation to Fe(CN)_6_^3−^, which releases an electron that travels *via* the external circuit to the cold electrode. There, it facilitates the reduction of Fe(CN)_6_^3−^ back to Fe(CN)_6_^4−^. Consistent with thermogalvanic behavior, a linear relationship between the steady-state voltage and temperature gradient is observed. The pure PAAm hydrogel electrolyte exhibited a *S*_e_ of 1.32 mV K^−1^, in agreement with previously reported values (Fig. S2).^15,31,36^ The introduction of EG or TMP solvents significantly influenced the thermopower, with Hybrid-E_30_T_0_ and Hybrid-E_0_T_30_ achieving *S*_e_ values of 2.0 mV K^−1^ and 2.38 mV K^−1^, respectively (Fig. S3). With higher organic solvent content, however, only marginal gains in *S*_e_ are achieved and a detrimental effect on ionic conductivity occurs. Hence, an additional increase in co-solvent concentration was not pursued in this study.

The *S*_e_ of the hybrid electrolyte system was optimized through rational design of co-solvent composition to effectively modulate the solvation environment of redox ions. We systematically varied the volume ratios of H_2_O, EG, and TMP while maintaining constant PAAm matrix content. Fig. 2b shows that *S*_e_ achieved a maximum of 2.52 mV K^−1^ with a solvent ratio of *V*_Water_ : *V*_EG_ : *V*_TMP_ = 7 : 0.7 : 2.3, *i.e.,* the Hybrid-E_7_T_23_ system. The ionic conductivity (*σ*) of the electrolytes was also significantly influenced by solvent composition. The pristine PAAm hydrogel exhibited *σ* = 5.48 S m^−1^, while that in single-solvent systems decreased to 1.86 S m^−1^ (Hybrid-E_30_T_0_) and 2.25 S m^−1^ (Hybrid-E_0_T_30_), as shown in Fig. S4. However, the rationally designed co-solvent system Hybrid-E_7_T_23_ restored conductivity to 4.01 S m^−1^. Our subsequent analysis suggests that single-solvent addition triggers selective precipitation of Fe(CN)_6_^4−^ ions, which enhances thermopower but compromises ionic conductivity. In contrast, the co-solvent approach modifies the solvation structure to facilitate ion transport while maintaining high *S*_e_.

The thermoelectrochemical performance of Hybrid-E_7_T_23_ was evaluated under varying Δ*T*. Fig. 2c depicts the measured output current–voltage–power curves when the cold electrode is maintained at 20 °C. As Δ*T* increased from 10 K to 40 K, both the open-circuit voltage (*V*_oc_) and short-circuit current density (*I*_sc_) increase steadily. The corresponding maximum output power (*P*_max_) and normalized power density (*P*_max_/Δ*T*^2^) are summarized in Fig. 2d. At Δ*T* = 40 K, *P*_max_ reached 821.4 mW m^−2^, with a normalized power density *P*_max_/Δ*T*^2^ of 0.513 mW m^−2^ K^−2^, in close agreement with theoretical predictions (Note S1, SI). Notably, this value exceeds those of single-solvent QTECs (0.07 and 0.16) by factors of 7.3 and 3.2, respectively.^29,31^ Further benchmarking against previous solvation-modulated QTECs without Gdm-based agent additives confirms significant improvements in both *S*_e_ and *P*_max_/Δ*T*^2^ (Fig. 2e and Table S2), thus confirming the efficacy of the co-solvent design strategy.

The thermal conductivity (*κ*) represents another parameter for quantifying heat-to-electricity conversion in QTECs.^37,38^ In contrast to conventional liquid electrolytes, a reduced thermal conductivity of 0.58 W m^−1^ K^−1^ occurs in PAAm hydrogel electrolyte due to restricted convection within its polymer network. The co-solvents strengthen hydrogen-bond networks that restrict molecular collisions and increases viscosity to suppresses internal microconvection.^39^ This contributes to further reduction of *κ* (0.443 W m^−1^ K^−1^) in the Hybrid-E_7_T_23_ electrolyte, a characteristic that favors the maintenance of a stable Δ*T* across the thermocell (Fig. S5). The thermoelectrochemical performance of Hybrid-E_7_T_23_ was further assessed based on its energy conversion efficiency (*η*) and Carnot-relative efficiency (*η*_r_). Based on the formulation provided in Note S2 (SI), the maximum *η* and *η*_r_ respectively reached 0.0695% and 1.04% under a Δ*T* of 40 K. Moreover, the Hybrid-E_7_T_23_ electrolyte demonstrates excellent moisture retention (Fig. S6), indicating strong potential for long-term operation. Thus, the assembled QTEC preserves approximately 95.6% of its initial performance, characterized by a high *S*_e_ of 2.41 mV K^−1^, even after ten days (Fig. 2g and S7).

As thermoelectrochemical conversion depends solely on Δ*T* rather than perceivable heat, we evaluated the electrolyte's heat-to-electricity conversion capability at sub-zero temperatures by fixing the hot electrode at 0 °C. When the cold side was cooled to −25 °C, the PAAm electrolyte showed severe performance degradation, yielding a *S*_e_ of 0.63 mV K^−1^ due to impeded ion transport and sluggish redox kinetics (Fig. 2h). In contrast, differential scanning calorimetry (DSC) revealed that the Hybrid-E_7_T_23_ electrolyte exhibits a freezing point of −41.8 °C, attributable to the co-solvent's disruption of the hydrogen-bond network in water (Fig. S8). Consequently, the Hybrid-E_7_T_23_ electrolyte maintained virtually unaffected thermoelectrochemical performance at sub-zero temperatures. In particular, a *S*_e_ of 2.47 mV K^−1^ was generated under the same conditions, a value that closely matches the room temperature data to exhibit a superb ∼97% retention (Fig. 2h). The excellent retention is corroborated by the robust ionic conductivity measured under the low-temperature conditions (Fig. S9). These results conclusively demonstrate the remarkable anti-freezing properties of our co-solvent QTEC, which allows for sustained thermal energy harvesting at sub-ambient temperatures.

### 2.3 Mechanism of enhanced thermoelectrochemical performance

The enhanced thermoelectrochemical performance of Hybrid-E_7_T_23_ can be elucidated *via* molecular-level interactions. At room temperature, Fe(CN)_6_^3−^ remains fully dissolved in hybrid TMP/EG solutions, whereas Fe(CN)_6_^4−^ readily precipitates (Fig. S10 and S11). The selective precipitation is also evident in hybrid solutions containing both Fe(CN)_6_^3−^ and Fe(CN)_6_^4−^ ions, as shown in Fig. S12. Ultraviolet-visible (UV-vis) spectra and optical photographs confirm that Fe(CN)_6_^4−^ exhibits negligible intrinsic solubility in pure EG solvent (Fig. S13). X-ray diffraction (XRD) analysis identified the precipitate formed in both pure EG solvent (Fig. S14) and hybrid TMP/EG solutions (Fig. S15) as primarily Fe(CN)_6_^4−^, with the characteristic peaks matching the standard powder diffraction file of K_4_Fe(CN)_6_. This difference in solubility, as documented in various solvent systems,^31,40^ arises from differential ion–solvent interactions between redox ions that mediate long-range ionic correlations. Furthermore, Fe(CN)_6_^4−^ exhibits pronounced thermosensitive solubility as shown in Fig. S16, which leads to spatial crystallization/dissolution under a temperature gradient. The total *S*_e_ in Hybrid-E_*x*_T_*y*_ is thus governed by both the solvation entropy difference and the concentration gradient established by the redox couple (Note S3).

**Fig. 3:**
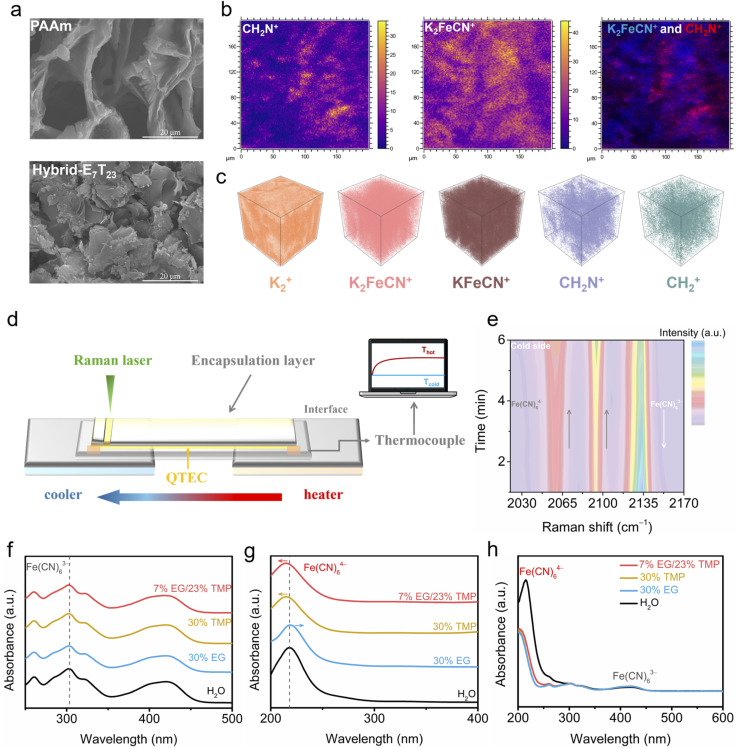
Characterization of electrolyte structures. (a) Morphological characterization of PAAm electrolyte and Hybrid-E_7_T_23_ electrolyte. (b) TOF-SIMS 2D spectra and (c) 3D reconstruction visual images of several secondary ion fragments in Hybrid-E_7_T_23_. (d) Schematic diagram of the *in situ* Raman measurement setup. (e) Real-time monitoring of the Fe(CN)_6_^4−/3−^ redox couple *via in situ* Raman spectroscopy for the cold side of Hybrid-E_7_T_23_ at Δ*T* = 10 K. UV-vis spectra of (f) Fe(CN)_6_^3−^, (g) Fe(CN)_6_^4−^, and (h) Fe(CN)_6_^4−/3−^ aqueous solutions with varying EG/TMP ratios.

To validate the formation of a concentration gradient within THs, we characterize the morphologies of the hydrogels using scanning electron microscopy (SEM). As shown in Fig. 3a and S17, the pure PAAm hydrogel exhibits a porous 3D network structure, whereas the surface of Hybrid-E_7_T_23_ is covered with abundant micrometer-sized crystals, likely resulting from the precipitation of Fe(CN)_6_^4−^ ions. This observation was further corroborated by Raman spectroscopy (Fig. S18). The crystallization is confined within the hydrogel network, which enables the establishment of a stable concentration gradient across Hybrid-E_7_T_23_. Furthermore, time-of-flight secondary ion mass spectrometry (TOF-SIMS) in positive ion mode revealed a complementary spatial distribution of CH_2_N^+^ (representing PAAm chains) and K_2_FeCN^+^ (derived from K_3_Fe(CN)_6_/K_4_Fe(CN)_6_) in Hybrid-E_7_T_23_ (Fig. 3b). The results indicate that the redox ions not only bind to the polymer matrix but also form crystalline phases within the hydrogel. Depth profiling (Fig. S20) was also applied to probe the distribution of components both on the surface and throughout the bulk of the hydrogel. The uniform distribution, as demonstrated from the resulting 3D reconstruction in Fig. 3c, ensures coherent ion transport pathways within the electrolyte.

During the operation of QTECs, the evolution of local ion concentrations under a Δ*T* was further examined using *in situ* Raman spectroscopy (Fig. 3d). Characteristic peaks at 2058 and 2093 cm^−1^ can be assigned to Fe(CN)_6_^4−^, while a peak at 2132 cm^−1^ corresponds to Fe(CN)_6_^3−^.^39,41^ In the PAAm electrolyte, the Fe(CN)_6_^3−^ peak intensity on the cold side decreased with prolonged heating, whereas the Fe(CN)_6_^4−^ signal increased (Fig. S21), which is indicative of Δ*T*-driven ion transport and redox cycling at the electrodes. In contrast, Hybrid-E_7_T_23_ exhibited similar but more pronounced concentration fluctuations under Δ*T*, with different rates of spectral change (Fig. 3e). This behavior results from the thermosensitive solubility of Fe(CN)_6_^4−^, where reversible crystallization/dissolution under Δ*T* sustains a stable concentration gradient that leads to additional entropy change of the redox reaction for thermopower enhancement.

To determine the relative contributions of solvation entropy differences *versus* concentration gradients to thermoelectrochemical performance, UV-vis spectroscopy was first employed to probe the ion–solvent interactions. Fig. 3f shows that as the EG/TMP ratio varied, the characteristic absorption peaks of Fe(CN)_6_^3−^ remained unchanged in the UV-vis spectra. In contrast, the absorption peak of Fe(CN)_6_^4−^ exhibited a noticeable shift from ∼218 nm as shown in Fig. 3g, indicating a stronger interaction between EG/TMP molecules and Fe(CN)_6_^4−^ compared to Fe(CN)_6_^3−^.^10,42,43^ These differential ion–solvent interactions not only reduce the solubility of Fe(CN)_6_^4−^ but are also believed to enlarge the difference in the solvation structure of redox ions.^21,31,44^ Furthermore, the comparative UV-vis analysis in Fig. 3h revealed that Fe(CN)_6_^4−^ concentration in three Hybrid-E_*x*_T_*y*_ electrolytes differs significantly from that in aqueous electrolytes, while Fe(CN)_6_^3−^ concentration remains largely unchanged. This signature correlates strongly with the observed thermosensitive solubility of Fe(CN)_6_^4−^ ions in solutions containing solvent molecules. The enhanced *S*_e_ in hybrid electrolytes, particularly Hybrid-E_30_T_0_ and Hybrid-E_0_T_30_, relative to pure hydrogel electrolytes can be attributed to concentration gradients. However, the similar concentration differences of Fe(CN)_6_^4−/3−^ ions are observed across various Hybrid-E_*x*_T_*y*_ electrolytes. To isolate the solvation entropy contribution, control hydrogel electrolytes denoted as Hybrid-E_*x*_T_*y*_ (sol) were prepared from the supernatant of equilibrated hybrid solutions. The *S*_e_ of Hybrid-E_7_T_23_ (sol) reaches 1.67 mV K^−1^, far exceeding those of the single solvent electrolytes (Fig. S20). Therefore, the superior *S*_e_ in Hybrid-E_7_T_23_ relative to Hybrid-E_30_T_0_ and Hybrid-E_0_T_30_ originates primarily from ion solvation entropy differences rather than concentration gradient effects.

**Fig. 4:**
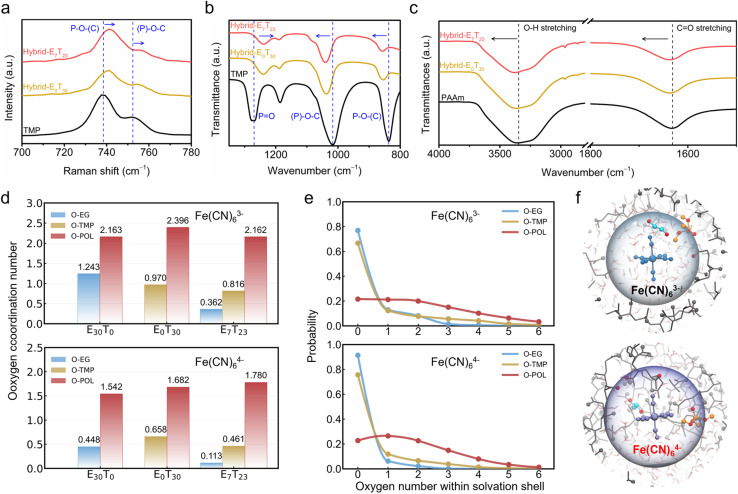
Effect of co-solvents on solvation of redox ions. (a) Raman spectra of various samples. (b) FTIR spectra of TMP solvent and various samples. (c) FTIR spectra of PAAm electrolyte and various samples. (d) Oxygen coordination number of Fe(CN)_6_^4−/3−^ ions from EG, TMP, and polymer side chains (POL). (e) Probability distribution of oxygen coordination numbers from EG, TMP and POL around Fe(CN)_6_^4−/3−^ ions. (f) Representative snapshots of the solvation shell structures of Fe(CN)_6_^3−^ and Fe(CN)_6_^4−^ ions in the Hybrid-E_7_T_23_ system. Color scheme: Fe(CN)_6_^4−/3−^ (blue), solvation shell (large transparent sphere), EG molecules (yellow), TMP molecules (orange), and polymer chains (gray). Oxygen atoms (from the three species) within the solvation shells are shown in red spheres. Hydrogen atoms from the three species are omitted for clarity. Water molecules are shown as transparent bonds.

The above analysis demonstrates that the co-solvent strategy achieves synergistic enhancement through two mechanisms: (1) concentration gradients that enhance performance relative to pure hydrogels; (2) solvation entropy differences that provide an improvement in Hybrid-E_7_T_23_ relative to single-solvent hydrogels. Next, spectroscopic analyses were performed to investigate the role of solvent in modulating solvation structures in hybrid electrolytes. Raman spectra of the electrolytes show that the P–O–C vibrational modes in pure TMP, located at 737.6 and 752.8 cm^−1^, shift noticeably in hybrid electrolytes (Fig. 4a). This is a consequence of interactions between TMP and other molecules or ions.^45–47^ FTIR spectra further confirm these interactions (Fig. 4b), with characteristic TMP peaks shifting significantly upon hybrid electrolyte formation. Furthermore, TMP addition caused a shift in the O–H vibrational peak, an effect that is amplified by EG (Fig. S23). These co-solvent–water interactions disrupt the hydrogen-bond network, which is consistent with the observed freezing point depression.^48,49^ The restructured hydrogen-bond network in Hybrid-E_*x*_T_*y*_ is further supported by FTIR as shown in Fig. 4c. A blueshift in the C=O vibration (1600–1700 cm^−1^) is also observed,^50^ likely due to modified polymer–ion coordination and solvation structures. These findings collectively indicate that co-solvents significantly alter the microscopic solvation structure in Hybrid-E_7_T_23_.

Molecular dynamics simulations were performed to elucidate the impact of the ion solvation structure on the solvation entropy difference. We quantified the composition of the solvation structure of redox ions *via* the number of coordinated oxygen atoms from different species in Hybrid-E_*x*_T_*y*_. Fig. 4d shows that more solvent molecules are in the solvation shell of Fe(CN)_6_^3−^ than Fe(CN)_6_^4−^, consistent with the stronger hydration of higher-valence ions. Owing to its higher dipole moment and Gutmann donor number relative to the EG,^51^ TMP exhibits stronger ion–solvent interactions toward Fe(CN)_6_^4−^ ions. The introduction of co-solvents reduces solvent coordination numbers relative to their single-solvent counterparts and the magnitudes of reduction are more significant for EG solvents. Moreover, the number of oxygen atoms from the PAAm backbone coordinating to redox ions shows opposite trends for Fe(CN)_6_^4−^ and Fe(CN)_6_^3−^ in Hybrid-E_*x*_T_*y*_ as compared to single-solvent electrolytes. The result indicates a synergistic co-solvent effect between EG and TMP that facilitates differential binding of PAAm sites to Fe(CN)_6_^4−/3−^ ions, a key factor in amplifying the solvation entropy difference. Unlike conventional solvation-engineering strategies that rely solely on bulk liquid-phase regulation,^10,12^ our system demonstrates the active contribution of the polymer matrix.

To obtain deeper molecular insights into the solvation environment, the distribution of oxygen atoms from EG, TMP, and polymer side chains of Hybrid-E_*x*_T_*y*_ was examined as shown in Fig. 4e. The probability distributions of oxygen from the three species, particularly from side chains, around Fe(CN)_6_^3−^ are consistently smoother and broader than Fe(CN)_6_^4−^. This corresponds to a more disordered and structurally complex solvation shell with greater structural variability of low valence ions. Typical snapshots of solvation shell structures of Fe(CN)_6_^4−/3−^ are depicted in Fig. 4f. Together with the oxygen coordination data, these observations highlight differential solvation structures between the redox ions that directly enhance the solvation entropy difference. Furthermore, ionic conductivity measurements reveal that Hybrid-E_7_T_23_ exhibits superior ion transport compared to single-solvent systems. To understand this improvement, we quantified the cation–anion pairing in the electrolyte (Fig. S27). The results point to a lower degree of K^+^ pairing to Fe(CN)_6_^4−/3−^ in the co-solvent system, a trend that correlates with the observed increase in ionic conductivity. The co-solvents thus also promote salt dissociation for both K_3_Fe(CN)_6_ and K_4_Fe(CN)_6_, which leads to higher population of free ions available for enhanced ionic conductivity.

### 2.4 QTEC durability and device application

**Fig. 5:**
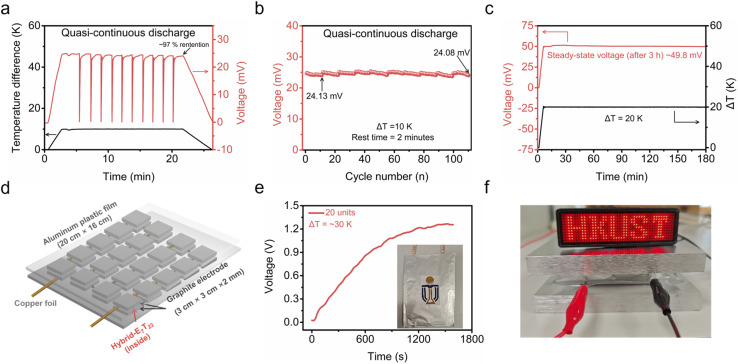
Potential application demonstration. (a) Voltage evolution along with the applied temperature difference in the quasi-continuous discharge process for the first 11 cycles. (b) Extended voltage life-span for 110 cycles at Δ*T* = 10 K. (c) Stability of the thermal voltage generated by Hybrid-E_7_T_23_ for 3 hours. (d) Schematic illustration of the integrated module with 20 coupled Hybrid-E_7_T_23_-based QTEC units. (e) Voltage–time curve and (f) the power electronic device for the module at Δ*T* = 30 K.

The QTEC operates continuously, as redox reactions proceed in opposite directions at the hot and cold electrodes while ionic transport replenishes reactants.^52^ To evaluate its operational stability, the lifespan of a Hybrid-E_7_T_23_-based QTEC was tested in the quasi-continuous discharge mode at room temperature with Δ*T* = 10 K. Fig. 5a shows the voltage over the first 11 cycles. Upon applying the temperature difference, the voltage increases and stabilizes at approximately 25 mV. The QTEC exhibits reproducible cycles of rapid discharge and recovery in the quasi-continuous discharge mode. The output performance in the 11th cycle (Fig. S28) shows a maximum power density of 40.2 mW m^−2^, close to the initial value of 45.5 mW m^−2^. To enable extended cycling, the thermocell was rested at room temperature (Δ*T* = 0 K) after every 11 charge–discharge cycles to allow recovery to its initial state. The excellent longevity was demonstrated by a stable thermovoltage of ∼24.1 mV even after 110 cycles (Fig. 5b). During this process, a minor voltage drop is also observed every 11 cycles due to polarization effects. The spontaneous voltage recovery arises from rapid redox ion diffusion that restores local concentration gradients at the electrode interfaces during the open circuit intervals. Furthermore, Hybrid-E_7_T_23_ maintains a stable voltage of ∼49.8 mV under a higher Δ*T* of 20 K as shown in Fig. 5c, demonstrating its potential for sustained heat-to-electricity conversion in a wide range of Δ*T*. Under a 2000 Ω load, the thermovoltage peaked and then decayed gradually, while the current density initially increased and then dropped (Fig. S29a). After 2 hours, the device still delivered ∼16.9 mV and ∼1.03 A m^−2^, yielding an energy density of 234 J m^−2^ (Fig. S29b).

As a harvester of low-grade waste heat, a single QTEC module typically generates insufficient output voltage for practical applications due to the small temperature gradients involved. Device integration is often needed to meet application requirements. We connected 20 individual Hybrid-E_7_T_23_-based QTEC units in series to construct an energy-harvesting device capable of generating useful voltages. As illustrated in Fig. 5d and S30, the device was fabricated by segmenting the Hybrid-E_7_T_23_ hydrogel electrolyte, using graphite plates as electrodes, and connecting the units with copper tape. The assembly was encapsulated in an aluminum plastic film to ensure operational stability by shielding it from external interference. When operated at a moderate Δ*T* of 30 K, the integrated device generated a high output voltage of ∼1.26 V, corresponding to a notable *S*_e_ of ∼42 mV K^−1^ (Fig. 5e). This performance demonstrates the device's ability to efficiently convert thermal energy into electricity. To assess its practical utility, the device was used to power electronic components. A light-emitting diode (LED) array was successfully illuminated under Δ*T* = 30 K (Fig. 5f). These results confirm the scalability of the Hybrid-E_7_T_23_ electrolyte and its practical potential for harvesting accessible low-grade waste heat in real-world applications.

## 3 Conclusion

In summary, we developed a co-solvent hydrogel electrolyte for QTECs that exhibits pronounced multiple synergetic effects for low-grade heat harvesting. The co-solvent hydrogel, composed of EG and TMP, leverages robust solvation ability to engineer an asymmetric solvation environment for the Fe(CN)_6_^4−/3−^ redox couple. This environment simultaneously enhances the solvation entropy difference and amplifies the concentration gradient of redox ions. The co-solvent-induced restructuring of PAAm coordination sites also promotes differential binding of Fe(CN)_6_^4−/3−^ ions. In addition, the co-solvents facilitate salt dissociation that favors continuum ion transport through the hydrogel. Beyond improving thermoelectrochemical properties, the reconfigured hydrogen-bond network endows the Hybrid-E_7_T_23_ electrolyte with anti-freezing capability and long-term stability. As a result, Hybrid-E_7_T_23_ delivers exceptional performance metrics: a *S*_e_ increased from 1.32 to 2.52 mV K^−1^, a normalized power density (*P*_max_/Δ*T*^2^) of 0.513 mW m^−2^ K^−2^, and sustained operation down to sub-zero conditions.

This study demonstrates that co-solvent interactions provide a viable strategy for tuning the solvation environment of redox ions in thermogalvanic hydrogels. The Hybrid-E_7_T_23_ system exemplifies this strategy, wherein maximizing the asymmetry between solvation structures resolves the trade-off between thermopower and ionic conductivity. Importantly, the performance enhancement is achieved through a simple, additive-free formulation. Our work presents fresh evidence for the principle that macroscopic properties can be engineered *via* molecular-level solvation control, thereby reinforcing its potential for the rational design of diverse high-performance, ion-conducting materials.

## Author contributions

Wentao Lin: conceptulization, investigation, data curation, formal analysis, writing – original draft, visualization; Shuo Niu: computations, formal analysis, writing-simulation results, visualization; Shukai Wu: methodology, validation; Chao Fang: conceptulization, supervision, analysis, visulization, writing – original draft, review, editing.

## Conflicts of interest

There are no conflicts to declare.

## Supplementary Material

SC-OLF-D6SC03942A-s001

## Data Availability

The data supporting this article have been included as part of the supplementary information (SI). Supplementary information is available. See DOI: https://doi.org/10.1039/d6sc03942a.

## References

[cit1] Shi X.-L., Zou J., Chen Z.-G. (2020). Advanced thermoelectric design: From materials and structures to devices. Chem. Rev..

[cit2] Liu Z., Sato N., Gao W., Yubuta K., Kawamoto N., Mitome M., Kurashima K., Owada Y., Nagase K., Lee C.-H., Yi J., Tsuchiya K., Mori T. (2021). Demonstration of ultrahigh thermoelectric efficiency of ∼7.3% in Mg_3_Sb_2_/MgAgSb module for low-temperature energy harvesting. Joule.

[cit3] Zhang L., Shi X.-L., Yang Y.-L., Chen Z.-G. (2021). Flexible thermoelectric materials and devices: From materials to applications. Mater. Today.

[cit4] Lheritier P., Torelló A., Usui T., Nouchokgwe Y., Aravindhan A., Li J., Prah U., Kovacova V., Bouton O., Hirose S., Defay E. (2022). Large harvested energy with non-linear pyroelectric modules. Nature.

[cit5] Yu B., Duan J. (2023). Electrochemical waste-heat harvesting. Science.

[cit6] Garofalo E., Bevione M., Cecchini L., Mattiussi F., Chiolerio A. (2020). Waste heat to power: Technologies, current applications, and future potential. Energy Technol..

[cit7] Liu C., Si Y., Zhang H., Wu C., Deng S., Dong Y., Li Y., Zhuo M., Fan N., Xu B., Lu P., Zhang L., Lin X., Liu X., Yang J., Luo Z., Das S., Bellaiche L., Chen Y., Chen Z. (2023). Low voltage–driven high-performance thermal switching in antiferroelectric PbZrO_3_ thin films. Science.

[cit8] Quickenden T. I., Mua Y. (1995). A review of power generation in aqueous thermogalvanic cells. J. Electrochem. Soc..

[cit9] Abraham T. J., MacFarlane D. R., Pringle J. M. (2013). High Seebeck coefficient redox ionic liquid electrolytes for thermal energy harvesting. Energy Environ. Sci..

[cit10] Duan J., Feng G., Yu B., Li J., Chen M., Yang P., Feng J., Liu K., Zhou J. (2018). Aqueous thermogalvanic cells with a high Seebeck coefficient for low-grade heat harvest. Nat. Commun..

[cit11] Duan J., Yu B., Huang L., Hu B., Xu M., Feng G., Zhou J. (2021). Liquid-state thermocells: Opportunities and challenges for low-grade heat harvesting. Joule.

[cit12] Inoue H., Zhou H., Ando H., Nakagawa S., Yamada T. (2024). Exploring the local solvation structure of redox molecules in a mixed solvent for increasing the Seebeck coefficient of thermocells. Chem. Sci..

[cit13] Cai X., Zeng Y., Liu P., Zhang Y., Wang L., Ke H., Long X., Jiang H., Yang W., Gan Z., Chen S., Duan J. (2026). Bioinspired ionic thermoreceptors with anisotropic architecture for thermotactile perception in robots. Sci. Adv..

[cit14] Meng H., Gao W., Chen Y. (2026). An ultrathin ionic thermoelectric cell design utilizing near body heat for self-powered wearable electronics. Nat. Commun..

[cit15] Zhang D., Zhou Y., Mao Y., Li Q., Liu L., Bai P., Ma R. (2023). Highly antifreezing thermogalvanic hydrogels for human heat harvesting in ultralow temperature environments. Nano Lett..

[cit16] Li Z., Xu Y., Zhang X. (2024). Progresses and insights of thermoelectrochemical devices for low-grade heat harvesting: From mechanisms, materials to devices. EnergyChem.

[cit17] Lin W., Wu S., Niu S., Hu Z., Chen G., Liu Z., Huang Y., Fang C. (2025). Critical design strategy of thermogalvanic hydrogels for low-grade heat harvesting. Adv. Sci..

[cit18] Lin Z., Hong J., Huang C., Zhang X., Shen S., Du Z., Zhou P., Miao Y.-B., Lin Z.-H., Lyu X., Zou Z. (2025). A strong, tough, and high-efficiency hydrogel thermocell for thermal energy harvesting. Nano Energy.

[cit19] Shin G., Baek J. Y., Kim J. H., Lee J. H., Kim H. J., So B. J., Choi Y., Yun S., Kim T., Jeon J. G., Kang T. J. (2025). Mechanically adaptable high-performance p(SBMA-MMA) copolymer hydrogel with iron (II/III) perchlorate for wearable thermocell applications. Adv. Funct. Mater..

[cit20] Chen Y., Huang Q., Liu T.-H., Qian X., Yang R. (2023). Effect of solvation shell structure on thermopower of liquid redox pairs. EcoMat.

[cit21] Li S., Li Z., Xu D., Hu R. (2024). Strong concentration gradient effect and weak solvation effect in thermopower enhancement in K_3_Fe(CN)_6_/K_4_Fe(CN)_6_ aqueous electrolyte with ethanol addition. Chem. Eng. J..

[cit22] Zeng Y., Yu B., Chen M., Zhang J., Liu P., Guo J., Wang J., Feng G., Zhou J., Duan J. (2025). Solvation entropy engineering of thermogalvanic electrolytes for efficient electrochemical refrigeration. Joule.

[cit23] Liu Y., Zhang S., Zhou Y., Buckingham M. A., Aldous L., Sherrell P. C., Wallace G. G., Ryder G., Faisal S., Officer D. L., Beirne S., Chen J. (2020). Advanced wearable thermocells for body heat harvesting. Adv. Energy Mater..

[cit24] Xu C., Sun Y., Zhang J., Xu W., Tian H. (2022). Adaptable and wearable thermocell based on stretchable hydrogel for body heat harvesting. Adv. Energy Mater..

[cit25] Zhang D., Mao Y., Ye F., Li Q., Bai P., He W., Ma R. (2022). Stretchable thermogalvanic hydrogel thermocell with record-high specific output power density enabled by ion-induced crystallization. Energy Environ. Sci..

[cit26] Lu X., Xie D., Zhu K., Wei S., Mo Z., Du C., Liang L., Chen G., Liu Z. (2023). Swift assembly of adaptive thermocell arrays for device-level healable and energy-autonomous motion sensors. Nano-Micro Lett..

[cit27] Gui J.-X., Cheng Y., Ren K., Liu Z.-P., Zhu Z., Xue Z.-Y., Zhu Y., Wang R.-H., Pei G., Sui J., Chen L.-F. (2025). Development of ternary hydrogel
electrolytes for superior gel thermocells: Exceptional anti-drying, anti-freezing, and mechanical robustness. Adv. Mater..

[cit28] Yu B., Duan J., Cong H., Xie W., Liu R., Zhuang X., Wang H., Qi B., Xu M., Wang Z. L., Zhou J. (2020). Thermosensitive crystallization–boosted liquid thermocells for low-grade heat harvesting. Science.

[cit29] Li J., Chen S., Han Z., Qu X., Jin M., Deng L., Liang Q., Jia Y., Wang H. (2023). High performance bacterial cellulose organogel-based thermoelectrochemical cells by organic solvent-driven crystallization for body heat harvest and self-powered wearable strain sensors. Adv. Funct. Mater..

[cit30] Liu L., Zhang D., Bai P., Mao Y., Li Q., Guo J., Fang Y., Ma R. (2023). Strong tough thermogalvanic hydrogel thermocell with extraordinarily high thermoelectric performance. Adv. Mater..

[cit31] Liu Z., Hu Y., Lu X., Mo Z., Chen G., Liu Z. (2024). Electrolyte engineering of quasi-solid-state thermocells for low-grade heat harvest at sub-zero temperatures. Adv. Energy Mater..

[cit32] Dupont M. F., MacFarlane D. R., Pringle J. M. (2017). Thermo-electrochemical cells for waste heat harvesting–progress and perspectives. Chem. Commun..

[cit33] Xu Y., Li Z., Li S., Zhang S., Zhang X. (2025). Reversibly tuning thermopower enabled by phase-change electrolytes for low-grade heat harvesting. Energy Environ. Sci..

[cit34] Yu H., Liu X., Li M., Zhang H., Wang Y., Zhu M., Luo S., Zhang X., Liu Z., Yang Y., Chen W., Hu Z., Wang K., Meng W., Huang Z., Liu Z., Huang Y. (2026). Constructing High-Power N-Type Thermocells via Fluoride-Mediated Imidazole–Iodine Coordination. Angew. Chem..

[cit35] Gao W., Lei Z., Zhang C., Liu X., Chen Y. (2021). Stretchable and freeze-tolerant organohydrogel thermocells with enhanced thermoelectric performance continually working at subzero temperatures. Adv. Funct. Mater..

[cit36] Shi X., Ma L., Li Y., Shi Z., Wei Q., Ma G., Zhang W., Guo Y., Wu P., Hu Z. (2023). Double hydrogen-bonding reinforced high-performance supramolecular hydrogel thermocell for self-powered sensing remote-controlled by light. Adv. Funct. Mater..

[cit37] Liu Y., Cui M., Ling W., Cheng L., Lei H., Li W., Huang Y. (2022). Thermo-electrochemical cells for heat to electricity conversion: from mechanisms, materials, strategies to applications. Energy Environ. Sci..

[cit38] Yu M., Li H., Li Y., Wang S., Li Q., Wang Y., Li B., Zhu K., Liu W. (2024). Ionic thermoelectric gels and devices: Progress, opportunities, and challenges. EnergyChem.

[cit39] Lu X., Mo Z., Liu Z., Hu Y., Du C., Liang L., Liu Z., Chen G. (2024). Robust, efficient, and recoverable thermocells with zwitterion-boosted hydrogel electrolytes for energy-autonomous and wearable sensing. Angew. Chem., Int. Ed..

[cit40] Tanaka Y., Wake A., Inoue D., Moritomo Y. (2024). Concentration gradient effect in liquid thermoelectric device composed of organic-solvent-added aqueous solution containing K_4_[Fe(CN)_6_]/K_3_[Fe(CN)_6_]. Jpn. J. Appl. Phys..

[cit41] Wang Y., Zhang Y., Xin X., Yang J., Wang M., Wang R., Guo P., Huang W., Sobrido A. J., Wei B., Li X. (2023). In situ photocatalytically enhanced thermogalvanic cells for electricity and hydrogen production. Science.

[cit42] Liu Y., Yin L., Chen S., Liu Y., Liu Q., Yang L., Li Y., Zhang Q., Huang Y. (2024). A hydrogel thermoelectrochemical cell with high self-healability and enhanced thermopower both induced by zwitterions. J. Mater. Chem. A.

[cit43] Liu L., Zhang D., Bai P., Fang Y., Guo J., Li Q., Ma R. (2025). Fatigue-resistant and super-tough thermocells. Nat. Commun..

[cit44] Wu H., Song J., Gao N., Pang X., Li Y., Xu Z., Yu X. (2025). High performance eutectogel-based thermocell with a wide operating temperature range through a water-containing deep eutectic solvent strategy. Chem. Eng. J..

[cit45] Lin W., Chen D., Yu J. (2024). Manipulating the ionic conductivity and interfacial compatibility of polymer-in-dual-salt electrolytes enables extended-temperature quasi-solid metal batteries. J. Colloid Interface Sci..

[cit46] Ni J., Cheng Q., Wang M., Liu S., Ji H., He Y., Qian T., Yan C., Lu J. (2024). Reshaping hydrogen bond network in aqueous-aprotic hybrid electrolyte to achieve highly selective ambient ammonia synthesis. Appl. Catal., B.

[cit47] Chen J., Lin W., Wu Z., Chen D., Law H. M., Ciucci F., Yu J. (2026). A coordination-reinforced and encapsulated polymer electrolyte for durable and safe Na-metal batteries. Energy Storage Mater..

[cit48] Dai B., Shi X., Pei X., Xu F., Zhao Y. (2024). Synergistic dual co-solvents hybrid electrolyte design enabling high-voltage flexible aqueous lithium-ion fiber batteries. Energy Storage Mater..

[cit49] Lin L., Shao Z., Liu S., Yang P., Zhu K., Zhuang W., Li C., Guo G., Wang W., Hong G., Wu B., Zhang Q., Yao Y. (2025). High-entropy aqueous electrolyte induced formation of water-poor Zn^2+^ solvation structures and gradient solid-electrolyte interphase for long-life Zn-metal anodes. Angew. Chem., Int. Ed..

[cit50] Hu X., Feng L., Xie A., Wei W., Wang S., Zhang J., Dong W. (2014). Synthesis and characterization of a novel hydrogel: salecan/polyacrylamide semi-IPN hydrogel with a desirable pore structure. J. Mater. Chem. B.

[cit51] Wu S., Luo Y., Niu S., Lin W., Fang C. (2025). AI-informed solvation engineering for thermogalvanic electrolytes with high thermopower. J. Phys. Chem. Lett..

[cit52] Hu R., Cola B. A., Haram N., Barisci J. N., Lee S., Stoughton S., Wallace G., Too C., Thomas M., Gestos A., Cruz M. E. d., Ferraris J. P., Zakhidov A. A., Baughman R. H. (2010). Harvesting waste thermal energy using a carbon-nanotube-based thermo-electrochemical cell. Nano Lett..

