# Supplementary material for: Engineering asymmetric solvation structures for synergistically boosted quasi-solid thermocells
Source: Chem Sci. 2026 Jun 22. Online ahead of print. doi: 10.1039/d6sc03942a (PMC13312817; doi:10.1039/d6sc03942a)
Supplement: SC-OLF-D6SC03942A-s001 [file SC-OLF-D6SC03942A-s001.pdf]

Supplementary Information (SI) for Chemical Science.
This journal is © The Royal Society of Chemistry 2026

*Electronic Supplementary Information for*

# Engineering Asymmetric Solvation Structures for Synergistically Boosted Quasi-Solid Thermocells

Wentao Lin[a], Shuo Niu[a], Shukai Wu[a], Chao Fang[a,*]

[a] Sustainable Energy and Environment Thrust, The Hong Kong University of Science and Technology (Guangzhou), Guangzhou, 511400, Guangdong, China

[*]Corresponding author:
chaofang@hkust-gz.edu.cn

# 1. Experimental section

## 1.1 Materials

Potassium ferricyanide ($K_3Fe(CN)_6$, ≥99.5%), potassium hexacyanoferrate ($K_4Fe(CN)_6 \cdot 3H_2O$, >99.0%), and Trimethyl phosphate (TMP, 98%) were purchased from Shanghai Macklin Biochemical Technology Co., Ltd (Shanghai, China). Acrylamide (AAm, ≥99.9%), Ammonium persulphate (APS, ≥96%), *N*,*N*'-methylenebisacrylamide (MBAA, ≥99%), Ethylene glycol (EG, ≥99.8%) were purchased from Shanghai Aladdin Biochemical Technology Co., Ltd (Shanghai, China). Graphite plates were purchased from Qingdao Baofeng Graphite Produce Co., Ltd. All chemicals were used as received without any further purification or treatment. Ultrapure deionized water was utilized in all experiments.

## 1.2 Preparation of thermogalvanic hydrogels

First, 6 g AAm monomer was dissolved in 10 mL of deionized water. Then, 30 mg of APS (as the initiator) and 16.5 mg of MBAA (as the chemical crosslinker) were added. The precursor solution was stirred and placed at room temperature for 1 hour, then poured into a polytetrafluoroethylene mold and reacted at 70 °C to form a pure polyacrylamide hydrogel.

Next, to prepare the thermogalvanic hydrogels, the above polyacrylamide hydrogel was immersed in a 0.4 M $Fe(CN)_6^{4-/3-}$ solution and EG/TMP solvent with different concentrations at 70 °C for 3 h. The co-solvents hybrid hydrogel electrolytes with different ratios were denoted as Hybrid-$E_xT_y$ ($x$ = 0~30 and $y$ = 0~30), as shown in Table S1. The thermogalvanic hydrogel prepared without any organic solvent molecules is named PAAm. During the solvent exchange, the concentration gradient between the interior and exterior of the hydrogel networks drove the diffusion of the redox couple ions into the hydrogels.

## 1.3 Thermoelectrochemical performance measurements

The quasi solid-state TEC (QTEC) was a sandwich structure of platinum (Pt)|Hybrid-$E_xT_y$|Pt, and two thermocouples were attached to both ends of the QTEC to monitor the

temperature difference ($\Delta T$). The Seebeck coefficient ($S_e$) of the QTEC was calculated from the slope of the thermoelectrochemical potential ($\Delta V$) versus $\Delta T$ curves ($S_e = -\frac{\Delta V}{\Delta T}$), recorded on a custom-built apparatus consisting of a cold plate, a hot plate, a temperature control system, and a digital sourcemeter (Keithley DAQ6510 Solon, Ohio, USA). To minimize the potential influence of gravity-driven effects, the cold and hot plates were arranged in a horizontal configuration during all measurements. Specifically, the QTEC was heated in steps, while the temperature of the cold electrode was maintained at approximately 20 °C. When the voltage ($V$) achieved a stable condition, the heating process continued. The $V$–$\Delta T$ curve was plotted using data points collected from at least 5 consecutive heating steps

The current ($I$)–$V$ curves were created using points measured from the open circuit voltage to 0 V, while the power ($P$)–$V$ curves were obtained by multiplying the current and voltage data, which are recorded by a digital sourcemeter (Keithley 2450 Solon, Ohio, USA). A quasi-continuous discharge was performed in the following steps: beginning state ($\Delta T = 0$ K), the thermal charge in an open circuit ($\Delta T > 0$ K), the thermal discharge in $I$–$V$ form ($\Delta T > 0$ K), thermally charged back to a high voltage ($\Delta T > 0$ K), and resting at $\Delta T = 0$ K.

The ionic conductivity ($\sigma$) of Hybrid-$E_xT_y$ was derived from the electrochemical impedance spectroscopy (EIS, PARSTAT3000A, Princeton Applied Research) which was recorded over a frequency range from 1 MHz to 0.1 Hz. The $\sigma$ was calculated using the equation, $\sigma = L/RA$, where L is the thickness of electrolyte, $R$ is the bulk resistance, and $A$ is the effective contact area.

**1.4 Material characterization**

The thermal conductivity of the thermogalvanic hydrogel was tested by the thermal conductivity analyzer (Hot Disk TPS2500S). Differential scanning calorimetry (DSC, TA DSC2500) was used to determine the freezing point at temperatures ranging from 20 °C to −80 °C. The analysis was conducted in a nitrogen atmosphere with a heating/cooling rate

of 10 °C min$^{-1}$. Morphologies were obtained by a scanning electron microscope (SEM, Hitachi SU3900) at an acceleration voltage of 10 kV. Time-of-flight secondary-ion mass spectrometry (TOF-SIMS) was carried out using TOF-SIMS equipment (IonTof M6) which included a SEM, using $Bi^{3+}$ as the primary ion source (30 keV) and $Cs^{2+}$ as the sputter source, in positive ion mode. X-ray photoelectron spectroscopy (XPS) of the thermogalvanic hydrogel was obtained by an X-ray photoelectron spectrometer (ULVAC-PHI, PHI VersaProbe4). Ultraviolet-visible spectra (UV-vis) was collected in a UV-vis spectrophotometer (PerkinElmer, LAMBDA 1050+) with wavelength range of 200 nm to 600 nm. Fourier transform infrared (FTIR) spectra were recorded on a FTIR spectrometer (Bruker, Vertex 70V + Hyperion II) in a vacuum environment with a frequency range of 400-4000 cm$^{-1}$. Raman spectroscopy was performed on a laser Raman spectrometer (Renishaw, inVia-Qontor) with a laser wavelength of 532 nm. For the in-situ Raman monitoring, the exposure time was set to 5 s with 2 accumulations per spectrum, and the time interval between the start of successive measurements was 60 s. X-ray diffraction (XRD, Malvern PANalytical) with Cu Kα radiation ($\lambda$=1.5406 Å) in the 2$\theta$ range of 10-60° was applied to characterize phases.

**1.5 Molecular dynamics simulation**

The components of the molecular dynamics (MD) simulation system are listed in **Table S3**. The PAAm (POL) gel framework was constructed in three dimensions by introducing inter-chain linkages and connecting the head and tail carbon atoms across periodic box boundaries, thereby preventing polymer chain aggregation. The initial network and the equilibrated structure after relaxation are shown in **Figure S24**. The visualization of PAAm chains highlights the structural features of both inter-chain linkages and intra-chain head-to-tail connections.

All organic components were modeled using the general AMBER force field (GAFF)[1]. The partial atomic charges for solvent molecules and polymer units are obtained from the RESP method via Gaussian and Antechamber packages. The force field

parameters for water molecules and redox ions are taken from previous literature.[2] The topology (itp) files were generated by Sobtop tool[3]. Equilibrium MD simulations were performed in the isothermal-isobaric (*NpT*) ensemble at 330 K and 1 bar using GROMACS package (version 2022.2)[4]. Temperature and pressure were respectively maintained via the velocity-rescale thermostat[5] and Berendsen barostat[6]. Each production run spanned 10 ns to ensure adequate sampling of ion solvation structures. Three independent simulations were conducted to improve the statistical reliability of solvation structure through trajectory averaging.

## 2. Supplementary Notes

### Note S1 Evaluation of Temperature-insensitive maximum power density

The normalized maximum power density ($P_{\max}/(\mathit{\Delta} T)^2$ ) is a crucial parameter frequently used to assess the output performance of QTEC. For the test of $P_{\max}/(\mathit{\Delta} T)^2$ of QTEC in this work, the distance between the two electrodes ($d$) was 1.5 cm. Record the current-voltage curve from 0 V to the open-circuit voltage ($V_{oc}$). The power-voltage curve is generated by multiplying the current ($I_{sc}$) and voltage values at each point. The normalized maximum power density is calculated using the following formula:

$$P_{max} = \frac{V_{oc} \times I_{sc}}{4} \qquad (S1)$$

$$\frac{P_{\max}}{\mathit{\Delta} T^2} = \frac{V_{oc} \times I_{sc}}{4\mathit{\Delta} T^2} \qquad (S2)$$

### Note S2 Evaluation of thermal energy conversion efficiency and Carnot-relative efficiency

The thermal energy conversion efficiency ($\eta$) of a QTEC can be expressed as:

$$\eta = \frac{P_{\max}}{P_{\text{heat}}} = \frac{P_{\max} \times d}{\kappa \times \Delta T} \qquad (S3)$$

where $P_{\text{heat}}$ is the heat input power density, and $\kappa$ represents the thermal conductivity.

The Carnot-relative efficiency ($\eta_r$) can be calculated using the following equation:

$$\eta_{\text{r}} = \frac{\eta}{\eta_{\text{c}}} = \frac{P_{\max} \times d \times T_{hot}}{\kappa \times \Delta T^2} \quad (S4)$$

$\eta_c$ denotes the Carnot efficiency, and $T_{hot}$ refers to the temperature on the hot side of the sample.

**Note S3 Theoretical analysis of the thermopower**

For a hypothetical redox reaction in a QTEC is described as:

$$A \Leftrightarrow ne^- + B \quad (S5)$$

where A and B represent redox couple in different valence state, and n is the number of electrons transferred, respectively.

Under a $\Delta T$, the $S_e$ originating from the thermogalvanic effect is related to the entropy difference ($\Delta S_{rc}$) of the redox ions and can be evaluated as:

$$S_{\text{e}} = \frac{(S_{\text{B}} + \bar{S}_{\text{B}}) - (S_{\text{A}} + \bar{S}_{\text{A}}) - n\bar{S}_{\text{e}}}{nF} \approx \frac{S_{\text{B}} - S_{\text{A}}}{nF} = \frac{\Delta S_{rc}}{nF} \quad (S6)$$

where $n$ is the number of electrons transferred, $F$ is the Faraday's constant, and $\bar{S}_{\text{e}}$ is transport entropy of electrons in the external circuit. $S_{\text{A}}$ and $S_{\text{B}}$ (along with their corresponding $\bar{S}_{\text{A}}$ and $\bar{S}_{\text{B}}$) respectively represent the partial molar entropies (Eastman entropies) of $A$ and $B$.

Moreover, the $S_{\text{e}}$ can also be determined by the ratio of the equilibrium potential difference between the hot and cold electrodes to the $\Delta T$, which is expressed as:

$$S_{\text{e}} = \frac{R}{nF\Delta T}\left[T_{\text{hot}} \ln \frac{\gamma_{\text{oH}}^{\text{A}}}{\gamma_{\text{rH}}^{\text{B}}} + T_{\text{hot}} \ln \frac{C_{\text{oH}}^{\text{A}}}{C_{\text{rH}}^{\text{B}}}\right] - \frac{R}{nF\Delta T}\left[T_{\text{cold}} \ln \frac{\gamma_{\text{oC}}^{\text{A}}}{\gamma_{\text{rC}}^{\text{B}}} + T_{\text{cold}} \ln \frac{C_{\text{oC}}^{\text{A}}}{C_{\text{rC}}^{\text{B}}}\right] \quad (S7)$$

Here, $\gamma_{\text{oH}}^{\text{A}}$, $\gamma_{\text{rH}}^{\text{B}}$, $\gamma_{\text{oC}}^{\text{A}}$, and $\gamma_{\text{rC}}^{\text{B}}$ are activity coefficients of redox ions A/B on the hot/cold electrode, while $C_{\text{oH}}^{\text{A}}$, $C_{\text{rH}}^{\text{B}}$, $C_{\text{oC}}^{\text{A}}$, and $C_{\text{rC}}^{\text{B}}$ are concentrations of redox ions A/B on the hot/cold sides, respectively.

The addition of co-solvents (EG and TMP) not only modifies the solvation entropy difference ($\Delta S_{solv}$) of the redox ions but also substantially alters their concentration

gradient. Therefore, the $S_\mathrm{e}$ of the Hybrid-$E_xT_y$ system originates from contributions of both the ion $\Delta S_{solv}$ and the concentration gradient ( $\Delta S_{conc}$ ), which can be comprehensively expressed as:

$$S_\mathrm{e} = \frac{\Delta S_{solv} + \Delta S_{conc}}{nF} \quad (S8)$$

To ensure clarity, $S_\mathrm{e}$ is consistently defined as a positive value throughout the main text.

## 3. Supplementary Figures

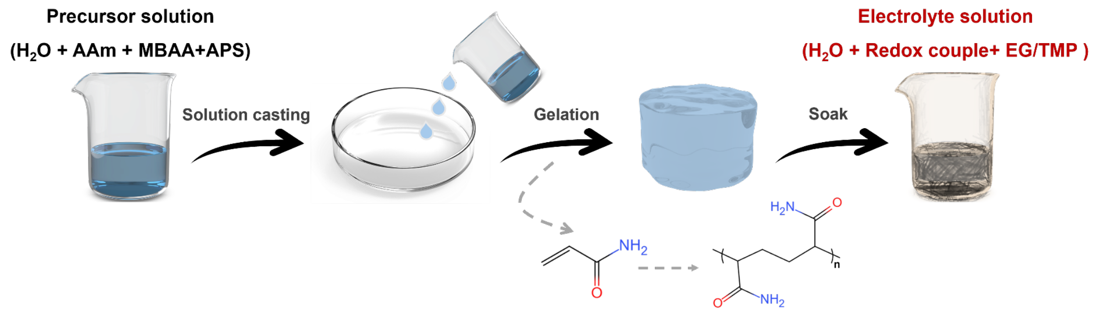


**Figure S1.** Schematic illustration of the preparation process of the Hybrid-$E_xT_y$.

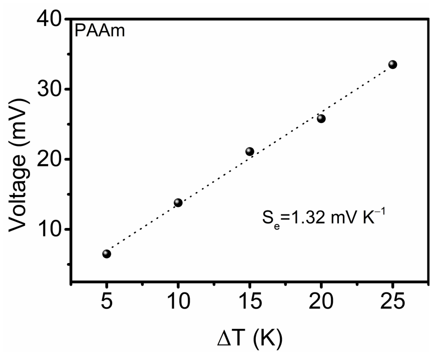


**Figure S2.** Fitted steady-state voltage−Δ$T$ plots for PAAm electrolyte.

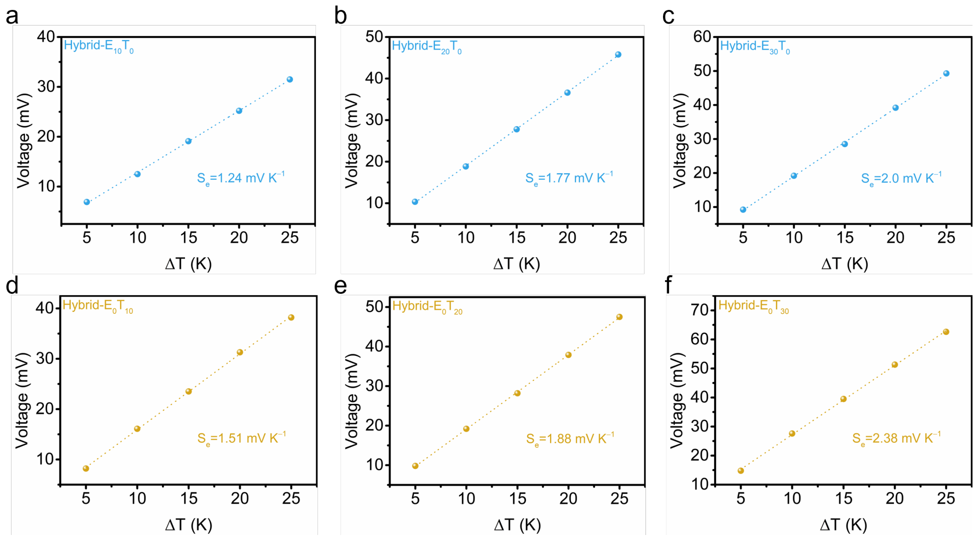


**Figure S3.** Fitted steady-state voltage−Δ$T$ plots for (a) Hybrid-$E_{10}T_0$, (b) Hybrid-$E_{20}T_0$, (c) Hybrid-$E_{30}T_0$, (d) Hybrid-$E_0T_{10}$, (e) Hybrid-$E_0T_{20}$, and (f) Hybrid-$E_0T_{30}$.

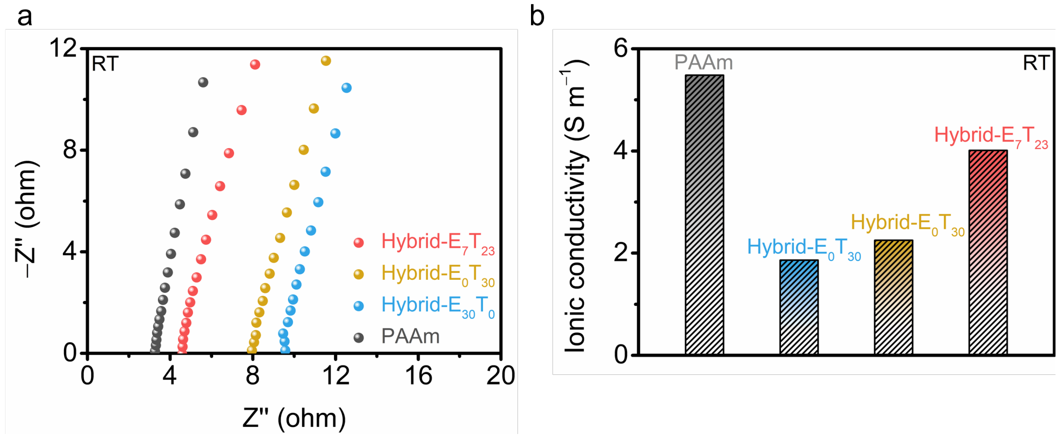


**Figure S4.** (a) Nyquist plots and (b) corresponding ionic conductivity of Hybrid-$E_xT_y$ at room temperature (RT).

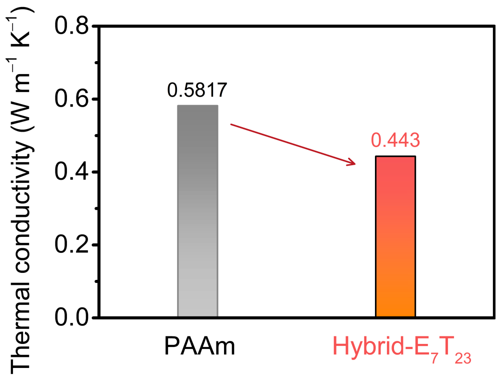


**Figure S5.** The thermal conductivity (κ) of PAAm electrolyte and Hybrid-$E_7T_{23}$ at 25 °C.

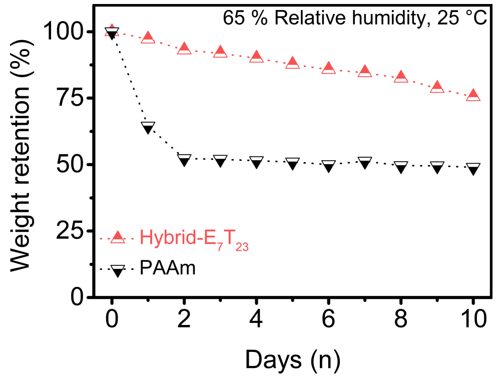


**Figure S6.** Time-dependent weight retention of PAAm electrolyte and Hybrid-$E_7T_{23}$ under ambient conditions.

The hydrogel electrolytes were stored at ambient conditions (25 °C, 65% relative humidity) without any packaging. As shown in Figure S6, the pure PAAm electrolyte underwent rapid water loss, drying out within two days. In contrast, the Hybrid-$E_7T_{23}$ exhibited a much slower water loss rate over the 10-day test period. This enhanced water retention capability is attributed to the multiple hydrogen bonding networks formed between PAAm, co-solvents, and water molecules. The mechanism will be discussed in detail later.

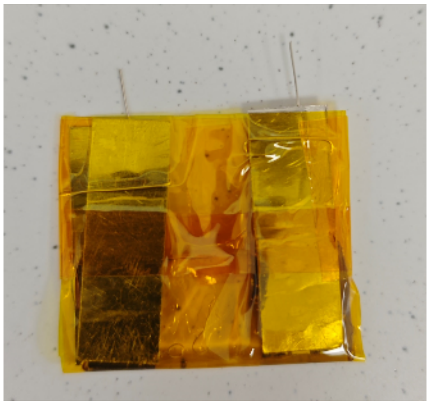

**Figure S7.** The photograph of the assembled platinum electrode/Hybrid-$E_7T_{23}$/platinum electrode QTEC device.

The QTEC was constructed by sandwiching the Hybrid-$E_7T_{23}$ hydrogel between two platinum electrodes. After assembly, the QTEC was encapsulated with two layers of polyimide tape and stored in air for 10 days, with its $S_e$ measured daily.

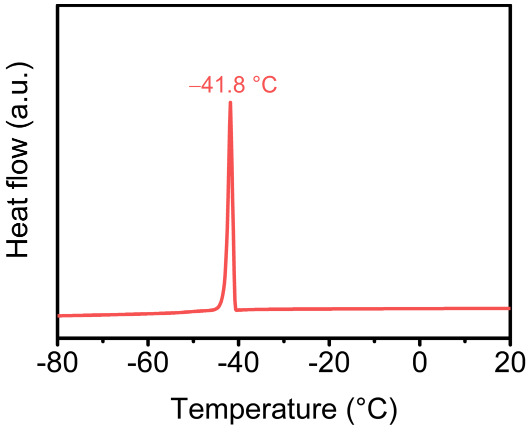


**Figure S8.** DSC curve of Hybrid-$E_7T_{23}$ measured with decreasing temperature from 20 to -80 °C.

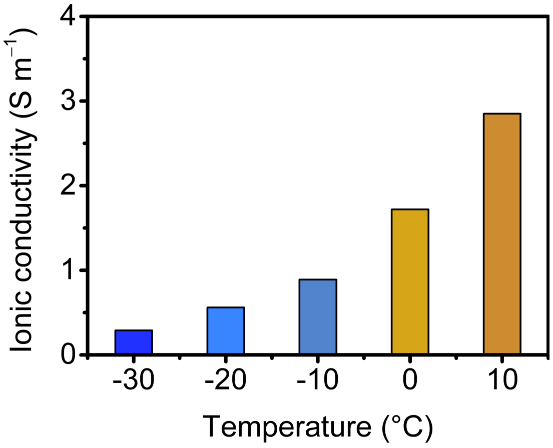


**Figure S9.** Ionic conductivity of Hybrid-$E_7T_{23}$ at low-temperatures.

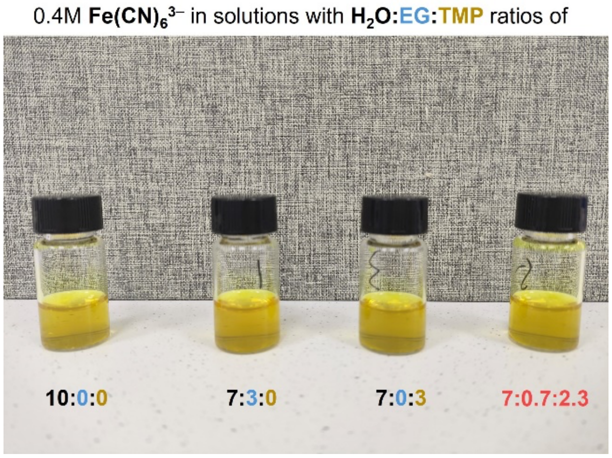


**Figure S10.** Photos of the 0.4 M $Fe(CN)_6^{3-}$ solutions with different $H_2O$:EG:TMP volume ratios.

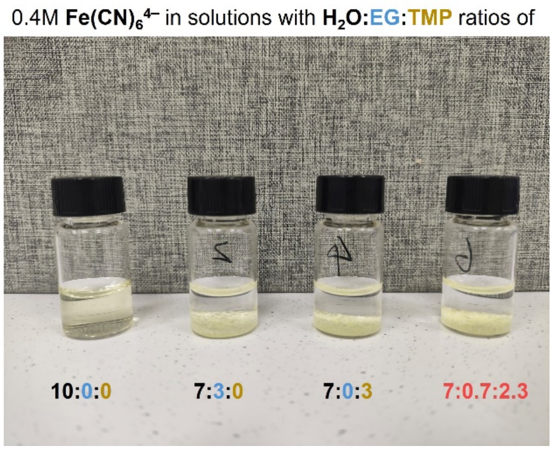


**Figure S11.** Photos of the 0.4 M $Fe(CN)_6^{4-}$ solutions with different $H_2O$:EG:TMP volume ratios.

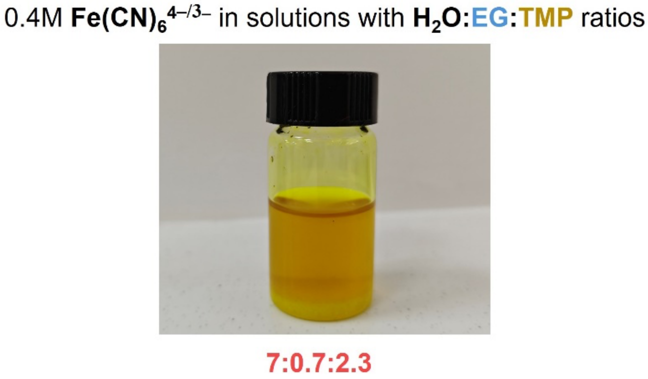


**Figure S12.** Photo of the 0.4 M $Fe(CN)_6^{4-/3-}$ solutions with $H_2O$:EG:TMP volume ratios of 7:0.7:2.3.

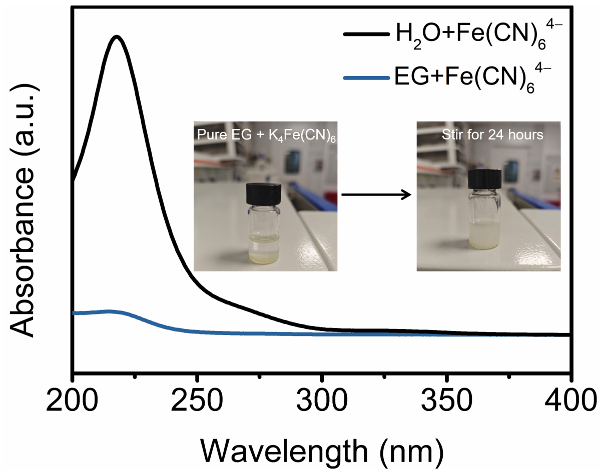


**Figure S13.** UV-Vis absorption spectra and dissolution behavior of $Fe(CN)_6^{4-}$ in pure EG solvent.

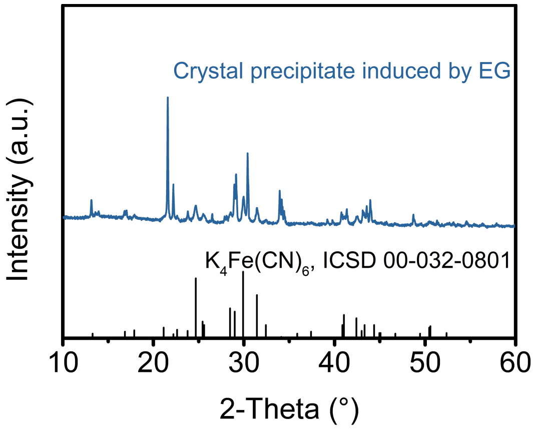


**Figure S14.** XRD spectra of crystal precipitates filtrated from the $Fe(CN)_6^{4-}$/pure EG solvent.

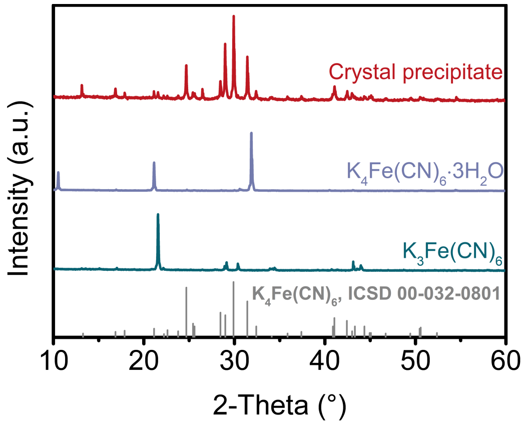


**Figure S15.** XRD spectra of pure $K_3Fe(CN)_6$, $K_4Fe(CN)_6 \cdot 3H_2O$, and crystal precipitates filtrated from the 0.4 M $Fe(CN)_6^{4-/3-}$ solutions with $H_2O$:EG:TMP volume ratios of 7:0.7:2.3.

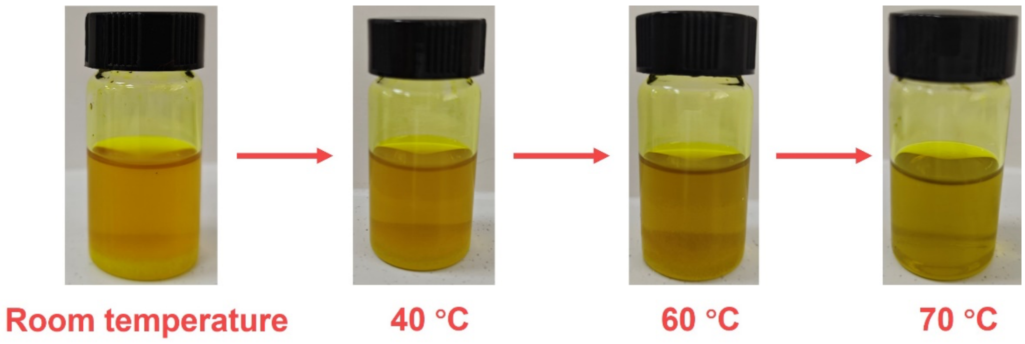


**Figure S16.** Photos of the 0.4 M $Fe(CN)_6^{4-/3-}$ solutions with $H_2O$:EG:TMP volume ratios of 7:0.7:2.3 at different temperatures.

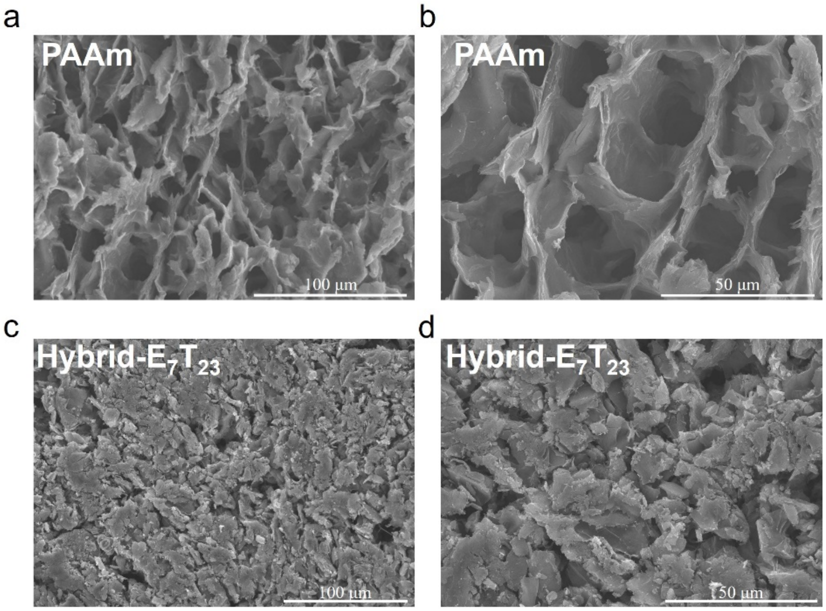


**Figure S17.** SEM images of various hydrogel electrolytes: (a-b) PAAm electrolyte and (c-d) Hybrid-$E_7T_{23}$.

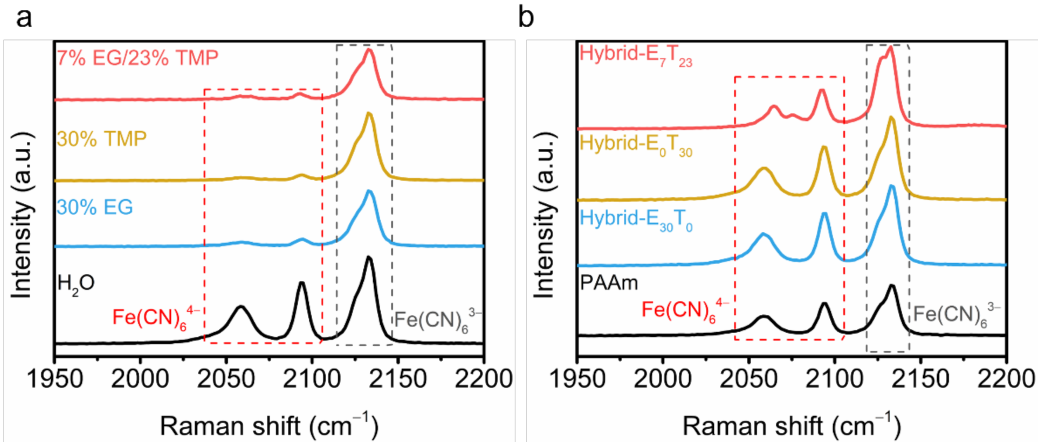


**Figure S18.** Raman spectra of (a) the $Fe(CN)_6^{4-/3-}$ liquid electrolytes with different solvent compositions and (b) various Hybrid-$E_xT_y$.

Raman spectra of the $Fe(CN)_6^{4-/3-}$ liquid electrolytes (LEs) reveal that the pure aqueous electrolyte has much stronger peaks at 2058 and 2093 cm$^{-1}$ (assigned to $Fe(CN)_6^{4-}$) than those with 30 vol% organic co-solvent, whereas the $Fe(CN)_6^{3-}$ peak at 2132 cm$^{-1}$ remains evident in all samples (Figure S18a). This suggests that the addition of EG/TMP

reduces the concentration of $Fe(CN)_6^{4-}$ in the LEs by inducing its precipitation. Conversely, in the Hybrid-$E_xT_y$, the characteristic $Fe(CN)_6^{4-}$ peaks are clearly observed (Figure S18b). This occurs since the EG/TMP-induced $Fe(CN)_6^{4-}$ precipitate is confined within hydrogel network, which results in pronounced scattering signatures.

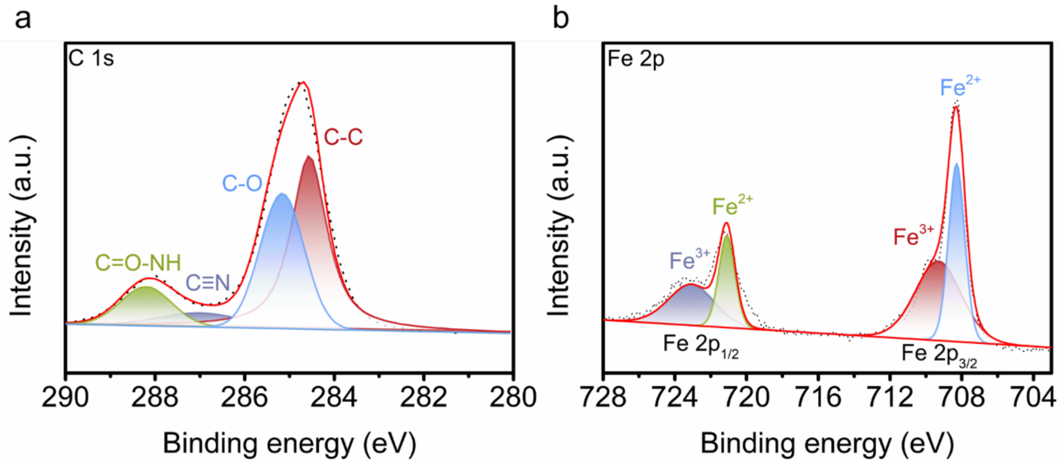


**Figure S19.** High-resolution XPS spectra (a) C 1s and (b) Fe 2p of Hybrid-$E_7T_{23}$.

The XPS spectra of Hybrid-$E_7T_{23}$ confirm the successful integration of the $Fe(CN)_6^{4-/3-}$ redox ions. The C 1s spectrum (Figure S19a) shows a distinct C≡N peak, while the Fe 2p spectrum (Figure S19b) clearly indicates the coexistence of $Fe^{2+}$ and $Fe^{3+}$ states. This demonstrates that the distinct oxidation states were maintained during hydrogel formation.

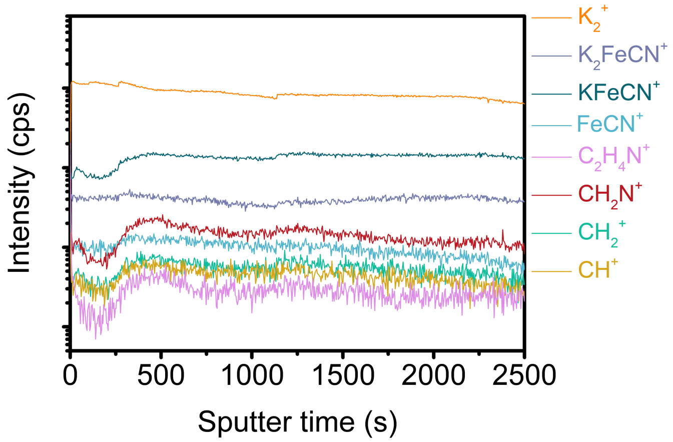


**Figure S20.** TOF-SIMS depth profiles of several secondary ion fragments in the Hybrid-$E_7T_{23}$.

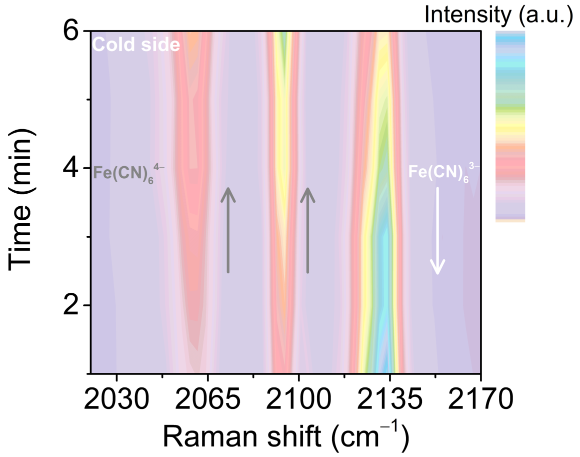


**Figure S21.** Real-time monitoring of $Fe(CN)_6^{4-/3-}$ redox couple via in-situ Raman for the cold side of PAAm electrolyte at $\Delta T = 10$ K.

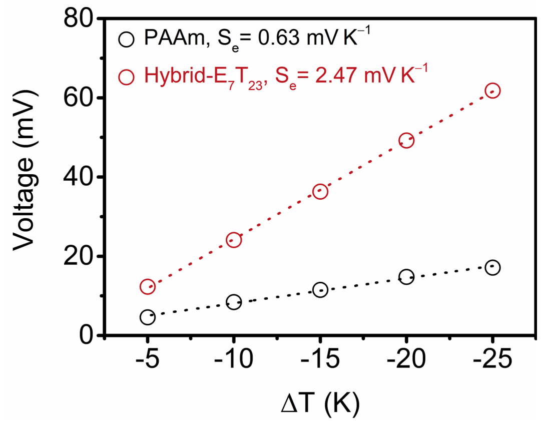


**Figure S22.** Fitted steady-state voltage−$\Delta T$ plots for Hybrid-$E_{30}T_0$ (sol), Hybrid-$E_0T_{30}$ (sol), and Hybrid-$E_7T_{23}$ (sol) electrolyte.

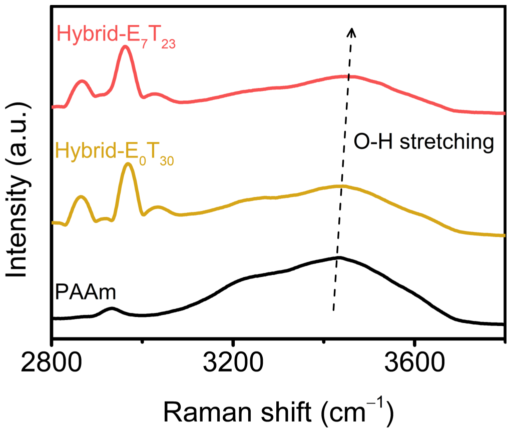


**Figure S23.** Raman spectra of PAAm electrolyte, Hybrid-$E_0T_{30}$, and Hybrid-$E_7T_{23}$ at range of 2800-3800 cm$^{-1}$.

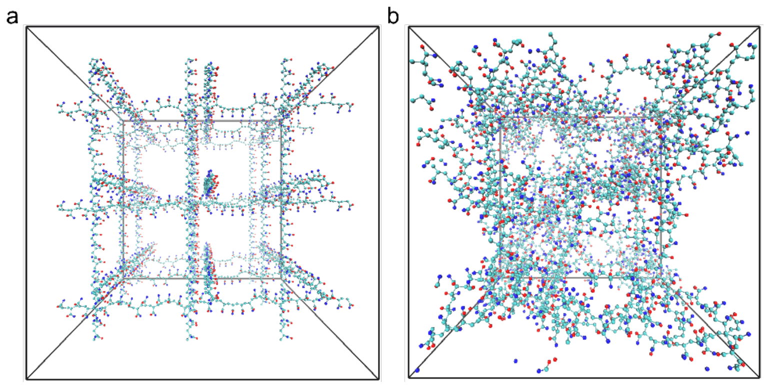


**Figure S24.** (a) Initial gel network and (b) stable gel network. Hydrogen atoms are omitted for clarity.

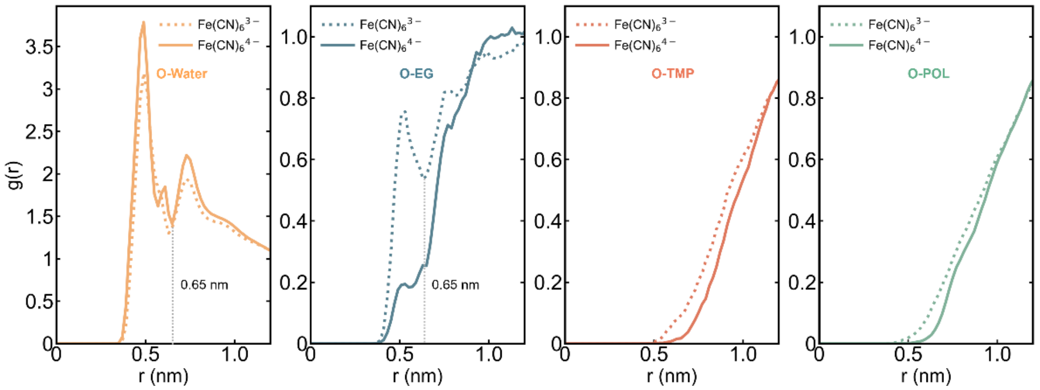


**Figure S25.** Radial distribution functions for oxygen atoms (from Water, EG, TMP and PAAm) around $Fe(CN)_6^{4-/3-}$ in Hybrid- $E_7T_{23}$ system.

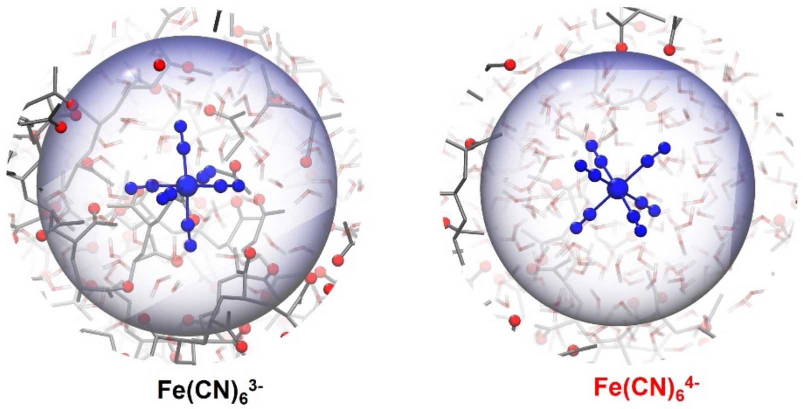


**Figure S26.** Representative snapshots of the solvation shell structures of $Fe(CN)_6^{3-}$ and $Fe(CN)_6^{4-}$ ions in PAAm electrolyte system. Color codes for atoms: Ions in blue, oxygen atom from polymer in red, polymer in grey, and solvation shell in large transparent sphere.

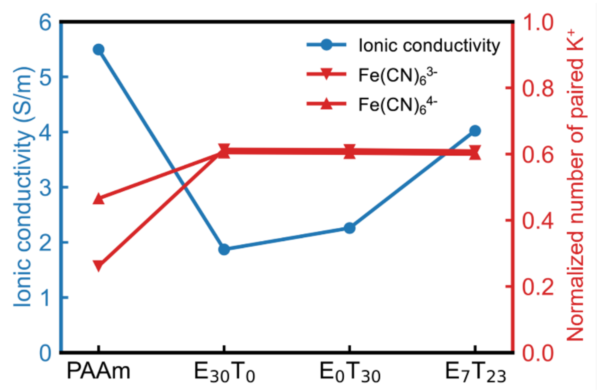


**Figure S27.** Ionic conductivity of different electrolytes and corresponding number of $K^+$ ions paired to the anion, which is normalized by the absolute valence of the anion.

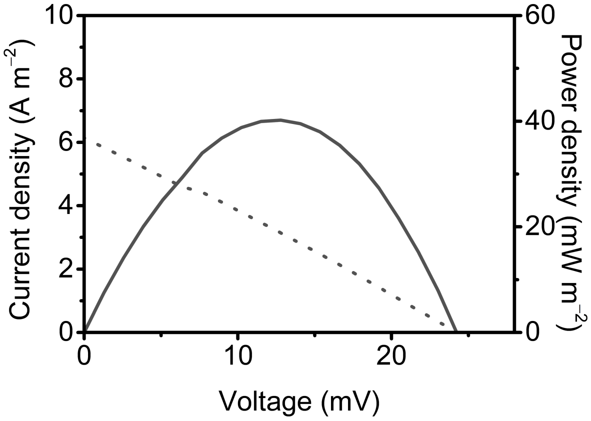


**Figure S28.** Output current–voltage–power curves of Hybrid-$E_7T_{23}$ for 11th charge/discharge cycle at $\Delta T = 10$ K.

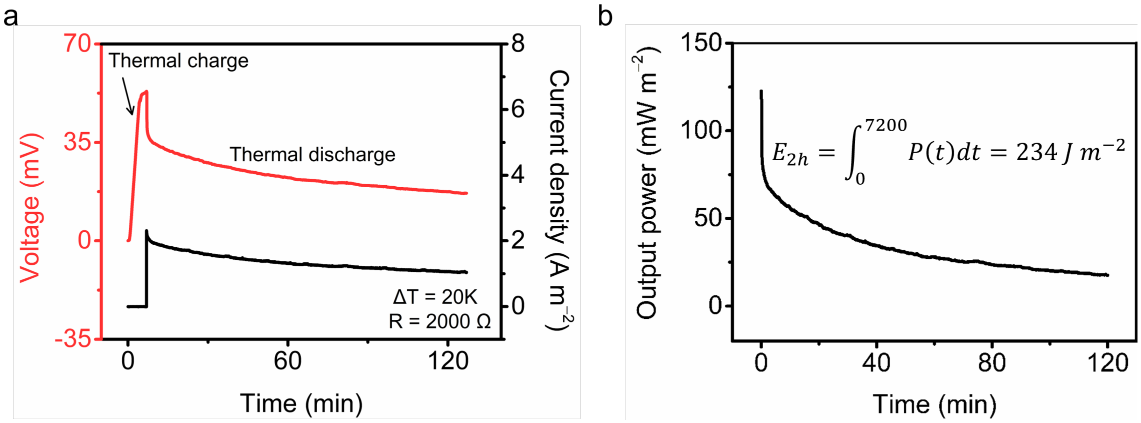


**Figure S29.** (a) Voltage and current curves for the continuous discharge process with an external resistance of 2000 Ω. (b) Output power density measured over 2 hours and corresponding generated energy density.

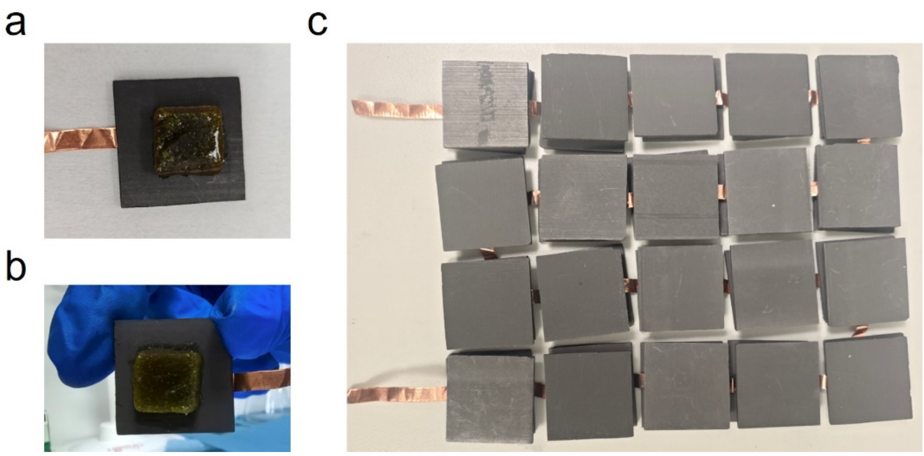


**Figure S30.** Optical photos of (a) Hybrid-$E_7T_{23}$ and graphite plate, (b) electrolyte- electrode adhesion test, and (c) Hybrid-$E_7T_{23}$-based QTEC device with 20 units.

## 4. Supplementary Tables

**Table S1** Formulated compositions to search for the maximum thermopower of Hybird-$E_xT_y$.

| Electrolyte | $H_2O$ (vol %) | TMP (vol %) | EG (vol %) | Thermopower (mV $K^{-1}$) |
|---|---|---|---|---|
| PAAm | 100 | 0 | 0 | 1.32 |
| Hybrid-$E_0T_{10}$ | 90 | 10 | 0 | 1.51 |
| Hybrid-$E_{10}T_0$ | 90 | 0 | 10 | 1.24 |
| Hybrid-$E_0T_{20}$ | 80 | 20 | 0 | 1.88 |
| Hybrid-$E_5T_{15}$ | 80 | 15 | 5 | 2.06 |
| Hybrid-$E_{10}T_{10}$ | 80 | 10 | 10 | 1.97 |
| Hybrid-$E_{15}T_5$ | 80 | 5 | 15 | 1.73 |
| Hybrid-$E_{20}T_0$ | 80 | 0 | 20 | 1.77 |
| Hybrid-$E_0T_{30}$ | 70 | 30 | 0 | 2.38 |
| Hybrid-$E_5T_{25}$ | 70 | 25 | 5 | 2.44 |
| Hybrid-$E_7T_{23}$ | 70 | 23 | 7 | 2.52 |
| Hybrid-$E_{10}T_{20}$ | 70 | 20 | 10 | 2.35 |
| Hybrid-$E_{13}T_{17}$ | 70 | 17 | 13 | 2.30 |
| Hybrid-$E_{15}T_{15}$ | 70 | 15 | 15 | 2.27 |
| Hybrid-$E_{17}T_{13}$ | 70 | 13 | 17 | 2.11 |
| Hybrid-$E_{20}T_{10}$ | 70 | 10 | 20 | 2.18 |
| Hybrid-$E_{25}T_5$ | 70 | 5 | 25 | 2.12 |
| Hybrid-$E_{30}T_0$ | 70 | 0 | 30 | 2.0 |

**Table S2** $S_e$ and $P_{max}/\Delta T^2$ of Hybrid-$E_7T_{23}$ thermogalvanic hydrogel developed in this work and those reported in the recent literature.

| Hydrogel | Redox couple | $S_e$ (mV $K^{-1}$) | $P_{max}/\Delta T^2$ (mW $m^{-2}K^{-2}$) | Ref. |
|---|---|---|---|---|
| PVA | $FeCl_{2/3}$ | 0.78 | 0.171 | [7] |
| PVA | $K_{3/4}Fe(CN)_6$ | 1.21 | 0.012 | [8] |
| 3FT-PVA | $K_{3/4}Fe(CN)_6$ | 1.5 | 0.22 | [9] |
| PAAm/AMPS | $K_{3/4}Fe(CN)_6$ | 1.5 | 0.61 | [10] |
| PAAm | $Fe(ClO_4)_{2/3}$ | 1.65 | 0.43 | [11] |
| p(SBMA-MMA) | $Fe(ClO_4)_{2/3}$ | 1.7 | 0.44 | [12] |
| PVA/starch/PDA-HNTs | $K_{3/4}Fe(CN)_6$ | 1.82 | 0.1825 | [13] |
| PAAm/EG/MXene | $K_{3/4}Fe(CN)_6$ | 2.02 | 0.16 | [14] |
| Gelatin/Betaine | $K_{3/4}Fe(CN)_6$ | 2.2 | 0.48 | [15] |
| BC/PG | $K_{3/4}Fe(CN)_6$ | 2.3 | 0.07 | [16] |
| Hybrid-$E_7T_{23}$ | $K_{3/4}Fe(CN)_6$ | 2.52 | 0.513 | This work |

PVA: Polyvinyl alcohol.

3-FT PVA: PVA after three cycles of unidirectional freezing training.

PAAm/AMPS: Polyacrylamide/2-acrylamide-2-methylpropane sulfonic acid.

p(SBMA-MMA): Sulfobetaine methacrylate/methyl methacrylate after polymerzation.

PVA/starch/PDA-HNTs: PVA/starch/polydopamine-halloysite nanotubes.

PAAm/EG/MXene: PAAm/ethylene glycol/$Ti_3C_2T_x$ MXene nanosheets.

BC/PG: Bacterial cellulose/propylene glycol

**Table S3** Components of different MD simulation systems.

| | $H_2O$ | EG | TMP | PAAm units | $Fe(CN)_6^{4-/3-}$ |
|---|---|---|---|---|---|
| PAAm electrolyte | 3857 | \ | \ | 972 | 28 |
| Hybrid-$E_{30}T_0$ | 2571 | 373 | \ | 972 | 28 |
| Hybrid-$E_0T_{30}$ | 2492 | \ | 180 | 972 | 28 |
| Hybrid-$E_7T_{23}$ | 2492 | 87 | 137 | 972 | 28 |

## References


1. Wang, J.; Wolf, R. M.; Caldwell, J. W.; Kollman, P. A.; Case, D., Development and testing of a general amber force field, *J. Comput. Chem.* **2004,** *25* (9), 1157-1174.
2. Chen, Y.; Huang, Q.; Liu, T.-H.; Qian, X.; Yang, R., Effect of solvation shell structure on thermopower of liquid redox pairs, *EcoMat* **2023,** *5* (9), e12385.
3. Tian, L., Sobtop, Version 1.0 (dev5), http://sobereva.com/soft/Sobtop (accessed on 3 April 2025).
4. Abraham, M. J.; Murtola, T.; Schulz, R.; Páll, S.; Smith, J. C.; Hess, B.; Lindahl, E., GROMACS: High performance molecular simulations through multi-level parallelism from laptops to supercomputers, *SoftwareX* **2015,** *1-2*, 19-25.
5. Bussi, G.; Donadio, D.; Parrinello, M., Canonical sampling through velocity rescaling, *J. Chem. Phys.* **2007,** *126* (1).
6. Hess, B.; Bekker, H.; Berendsen, H. J. C.; Fraaije, J. G. E. M., LINCS: A linear constraint solver for molecular simulations, *J. Comput. Chem.* **1997,** *18* (12), 1463-1472.
7. Liu, Y.; Zhang, S.; Zhou, Y.; Buckingham, M. A.; Aldous, L.; Sherrell, P. C.; Wallace, G. G.; Ryder, G.; Faisal, S.; Officer, D. L.; Beirne, S.; Chen, J., Advanced wearable thermocells for body heat harvesting, *Adv. Energy Mater.* **2020,** *10* (48), 2002539.
8. Yang, P.; Liu, K.; Chen, Q.; Mo, X.; Zhou, Y.; Li, S.; Feng, G.; Zhou, J., Wearable thermocells based on gel electrolytes for the utilization of body heat, *Angew. Chem. Int. Ed.* **2016,** *55* (39), 12050-12053.
9. Gao, W.; Lei, Z.; Chen, W.; Chen, Y., Hierarchically anisotropic networks to decouple mechanical and ionic properties for high-performance quasi-solid thermocells, *ACS Nano* **2022,** *16* (5), 8347-8357.
10. Lei, Z.; Gao, W.; Wu, P., Double-network thermocells with extraordinary toughness and boosted power density for continuous heat harvesting, *Joule* **2021,** *5* (8), 2211-2222.
11. Xu, C.; Sun, Y.; Zhang, J.; Xu, W.; Tian, H., Adaptable and wearable thermocell based on stretchable hydrogel for body heat harvesting, *Adv. Energy Mater.* **2022,** *12* (42), 2201542.
12. Shin, G.; Baek, J. Y.; Kim, J. H.; Lee, J. H.; Kim, H. J.; So, B. J.; Choi, Y.; Yun, S.; Kim, T.; Jeon, J. G.; Kang, T. J., Mechanically adaptable high-performance p(SBMA-MMA) copolymer hydrogel with iron (II/III) perchlorate for wearable thermocell applications, *Adv. Funct. Mater.* **2024,** *35* (12), 2412524.
13. Li, N.; Wang, Z.; Yang, X.; Zhang, Z.; Zhang, W.; Sang, S.; Zhang, H., Deep-learning-assisted thermogalvanic hydrogel E-skin for self-powered signature recognition and biometric authentication, *Adv. Funct. Mater.* **2024,** *34* (18), 2314419.

14. Liu, Z.; Hu, Y.; Lu, X.; Mo, Z.; Chen, G.; Liu, Z., Electrolyte engineering of quasi-solid-state thermocells for low-grade heat harvest at sub-zero temperatures, *Adv. Energy Mater.* **2024,** *14* (42), 2402226.
15. Lu, X.; Mo, Z.; Liu, Z.; Hu, Y.; Du, C.; Liang, L.; Liu, Z.; Chen, G., Robust, efficient, and recoverable thermocells with zwitterion-boosted hydrogel electrolytes for energy-autonomous and wearable sensing, *Angew. Chem. Int. Ed.* **2024,** *63* (29), e202405357.
16. Li, J.; Chen, S.; Han, Z.; Qu, X.; Jin, M.; Deng, L.; Liang, Q.; Jia, Y.; Wang, H., High performance bacterial cellulose organogel-based thermoelectrochemical cells by organic solvent-driven crystallization for body heat harvest and self-powered wearable strain sensors, *Adv. Funct. Mater.* **2023,** *33* (46), 2306509.
